# Protein ensemble modeling and analysis with MMMx

**DOI:** 10.1002/pro.4906

**Published:** 2024-02-15

**Authors:** Gunnar Jeschke

**Affiliations:** ^1^ Department of Chemistry and Applied Biosciences ETH Zürich Zürich Switzerland

**Keywords:** ensemble comparison, ensemble modeling, intrinsic disorder, weak order

## Abstract

Proteins, especially of eukaryotes, often have disordered domains and may contain multiple folded domains whose relative spatial arrangement is distributed. The MMMx ensemble modeling and analysis toolbox (https://github.com/gjeschke/MMMx) can support the design of experiments to characterize the distributed structure of such proteins, starting from AlphaFold2 predictions or folded domain structures. Weak order can be analyzed with reference to a random coil model or to peptide chains that match the residue‐specific Ramachandran angle distribution of the loop regions and are otherwise unrestrained. The deviation of the mean square end‐to‐end distance of chain sections from their average over sections of the same sequence length reveals localized compaction or expansion of the chain. The shape sampled by disordered chains is visualized by superposition in the principal axes frame of their inertia tensor. Ensembles of different sizes and with weighted conformers can be compared based on a similarity parameter that abstracts from the ensemble width.

## 1 INTRODUCTION

Intrinsically disordered regions (IDRs) are widespread among eukaryotic proteins, with the proportion of disordered residues in the human proteome estimated at 37–50% (Oates et al., 2012). While efficient and accurate prediction of folded domain structure has become possible with the AlphaFold2 (AF2) machine learning approach (Jumper et al., 2021), AF2 models for 95% of the human proteome (Tunyasuvunakool et al., 2021) have also shown that most human proteins have IDRs and that current machine learning models cannot predict the overall structure of these proteins (Ruff & Pappu, 2021). In particular, important signaling proteins tend to have a highly dynamic and modular architecture that allows for cooperative binding to multiple partners and allosteric transitions (Eustermann et al., 2015). Analogous considerations apply to many RNA‐binding proteins (Pereira et al., 2017). In these cases, binding events can cause partial disorder‐to‐order transitions that can only be described by ensemble models that quantify the extent and character of disorder (Bonomi et al., 2016; Bottaro & Lindorff‐Larsen, 2018; Hummer & Köfinger, 2015). It has been argued that we need quantitative descriptions of conformational ensembles in terms of distribution functions for the distances between residues (Ruff & Pappu, 2021). Such descriptions can be derived by combining electron paramagnetic resonance (EPR) techniques with site‐specific spin labeling (Jeschke, 2012a). Combined with spin‐label modeling using rotamer libraries (Polyhach et al., 2011; Polyhach & Jeschke, 2010), this approach allows the derivation of distributions of distances between residues. These are related to projections of the protein's energy landscape and provide valuable information about the width of the conformational ensemble (Jeschke, 2022). Since EPR techniques such as double electron–electron resonance (DEER; Schiemann et al., 2021) cover relevant length scales between 20 and about 100 Å and can be applied without size restrictions of the protein, they can go beyond the previously pursued approach (Ruff & Pappu, 2021) of detaching IDRs from their context and studying them as autonomous entities.

We recently introduced the MMMx toolbox (Jeschke & Esteban‐Hofer, 2022) that enables integrative ensemble modeling pipelines for intrinsically disordered proteins (IDPs; Esteban‐Hofer et al., 2023) and for IDRs bound to a single folded domain (Ritsch et al., 2021, 2022). MMMx can also model proteins with multiple folded domains connected by IDR linkers (Dorn et al., 2023). The toolbox is designed to use distance distribution restraints obtained through DEER experiments as the primary source of information on ensemble width (Jeschke, 2022). Thus, it complements existing ensemble modeling software such as ASTEROIDS (Salmon et al., 2010), FlexibleMeccano (Ozenne, Bauer, et al., 2012), PLUMED‐ISDB (Bonomi & Camilloni, 2017), and BioEn (Hummer & Köfinger, 2015). DEER studies require a substantial amount of effort for double mutant generation, spin labeling, and distance distribution measurements. It is therefore important to select optimal labeling positions. Such optimal choice can also improve the robustness and quality of ensemble models (Dorn et al., 2023). Here, we introduce features of MMMx for improved design of experiments. An important issue in such experimental studies is the comparison of ensembles obtained with different restraint sets or with different modeling approaches (Aina et al., 2023; Brüschweiler, 2003; Dorn et al., 2023; González‐Delgado et al., 2023; Huihui & Ghosh, 2020, 2021; Lazar et al., 2020; Lindorff‐Larsen & Ferkinghoff‐Borg, 2009; Tiberti et al., 2015). Here, we introduce a single‐valued similarity measure that abstracts from the ensemble width. The ensemble analysis module of MMMx complements existing toolkits such as PENSA (Vögele et al., 2022), ProDy (Zhang et al., 2021), or EnGens (Conev et al., 2023), which aim to summarize the information from ensemble structures or molecular dynamics (MD) trajectories into simpler descriptors of the conformational landscape. For example, the interpretation of ensemble models of IDPs and IDRs requires the detection of weak deviations from a polymeric random coil (Alston et al., 2023; Ritsch et al., 2021). Here, we extend the previously introduced (Jeschke, 2021; Jeschke & Esteban‐Hofer, 2022) site‐specific and section‐specific features for characterizing weak order and heterogeneous chain elongation and illustrate their utility with examples from the Protein Ensemble Database (PED; Ghafouri et al., 2023; Lazar et al., 2021).

This article is organized as follows. First, we introduce the ensemble representation used in MMMx and introduce a single‐valued similarity measure based on the mean square deviation of the distance in an abstract conformer space. Second, we discuss site‐specific measures of disorder, which have either a local or a longer‐range character. Third, we extend our approach (Jeschke, 2021; Ritsch et al., 2021) to characterize weak deviations from random coil behavior to a representation that is particularly sensitive to heterogeneous chain elongation. Fourth, we introduce the partitioning of a protein into folded domains and IDRs based on AF2‐predicted aligned error matrices. We compare this approach with partitioning based on one‐dimensional disorder prediction. Fifth, we discuss the choice of optimal spin labeling sites. Finally, we validate and illustrate some of the newly introduced features of MMMx. We conclude with a brief discussion of open issues in ensemble modeling and analysis.

## 2 RESULTS AND DISCUSSION

### 2.1 Ensemble representation

MMMx represents an ensemble with *C* conformers (index *c*) by *C* atomistic structures and a vector ***w*** of conformer weights *w*_*c*_. The weights are normalized, that is, they add up to one. To compare the information content of different ensembles for the same protein, we consider the Shannon entropy, which we define with a decadic logarithm

(1) $$s_{\text{Shannon}}=-\sum_{c}w_{c}\log_{10}w_{c.}$$

Of two ensembles that fit the experimental data equally well, the one with the lower Shannon entropy provides a more compact description. The representation of the data by such a parsimonious ensemble reduces the computational effort for further analysis and visualization and is therefore preferred in MMMx. However, we point out that parsimonious ensembles can be unrealistically small and narrow, especially if they are only determined by ensemble average constraints. MMMx is intended for modeling based on distance distributions (Jeschke, 2022) and such information largely protects against unrealistic ensemble contraction. In general, the choice between maximum parsimony, maximum entropy, or maximum occupancy approaches (Ravera et al., 2016) is an open question in ensemble reweighting (Gama Lima Costa & Fushman, 2022; Orioli et al., 2020).

To analyze the chain flexibility, it is sufficient to consider the backbone atoms N, Cα, and C of each residue, which define the backbone dihedral angles ϕ_*i*_ and ψ_*i*_ of a residue with index *i*. For the comparison of conformers and the application of concepts from polymer physics, the representation can be further reduced to Cα traces with the same weights *w*_*c*_. The distance root mean square deviation (DRMSD) between two conformers (indices *c*_1_ and *c*_2_) is invariant under affine transformations of the individual conformers. Two conformers with similar overall shape have low DRMSD. For a protein with *N* residues, DMRSD is given by

(2) $$D_{c_{1}c_{2}}=\sqrt{\frac{1}{N\left(N-1\right)/2}\sum_{i=1}^{N-1}\sum_{j>i}^{N}{\left(r_{i,j,c_{1}}-r_{i,j,c_{2}}\right)}^{2},}$$

where $r_{i,j,c_{1}}$ and $r_{i,j,c_{2}}$ are the distances between the Cα atoms of residues *i* and *j* in the conformers *c*_1_ and *c*_2_ respectively. $D_{c_{1}c_{2}}$ is non‐negative and symmetric and becomes zero if the two conformers are identical except for an affine transformation. As a metric induced by a norm, it satisfies the triangle inequality. Therefore, $D_{c_{1}c_{2}}$ can be interpreted as a distance in an abstract Euclidean conformer space whose dimension is not known a priori. The $D_{c_{1}c_{2}}$ can be arranged in a distance matrix ***D***. Clustering of conformers based on ***D*** is similar in spirit to clustering based on other pairwise Euclidean distances proposed earlier (Alston et al., 2023; Conev et al., 2023). Distance geometry methods allow embedding ***D*** in the conformer space and thus representing the set of conformers in this abstract space. Even without explicit embedding, we can define a mean square distance in conformer space for two ensembles of the same protein (indexed by *k* and *l*), which is averaged over all pairs of conformers,

(3) $${∆}_{\mathrm{kl}}=\sqrt{\sum_{c_{k}=1}^{C_{k}}\sum_{c_{l}=1}^{C_{l}}w_{k,c_{k}}w_{l,c_{l}}D_{c_{k}c_{l}}^{2}}$$

Here, we have arranged all conformers from both ensembles in a common distance matrix ***D*** and ***w***_*k*_ and ***w***_*l*_ are weight vectors for the individual ensembles. We note that the width of a single ensemble with index *k* is denoted by Δ_*kk*_. The latter parameter is zero if the ensemble consists of a single conformer or if all conformers in the ensemble are identical. Otherwise, it is greater than zero, which means that Δ_*kl*_ is not a Euclidean distance between the ensembles *k* and *l*. However, since Δ_*kl*_ is a measure of the mean difference between conformers in the two ensembles, we can define a dimensionless similarity measure *s*_*kl*_ as

(4) $$s_{\mathrm{kl}}=\frac{\sqrt{\Delta_{\mathrm{kk}}\Delta_{\mathrm{ll}}}}{\Delta_{\mathrm{kl}}}$$

If each ensemble consists of a single conformer and the two conformers are different, then *s*_*kl*_ = 0. If the two ensembles are identical, then Δ_*kk*_ = Δ_*ll*_ = Δ_*kl*_ and thus *s*_*kl*_ = 1.

### 2.2 Site‐specific measures of disorder

A site‐specific order parameter can be defined by a reinterpretation of the Flory characteristic ratio that we introduced earlier (Jeschke & Esteban‐Hofer, 2022). For this purpose, we consider Cα‐Cα vectors ***r***_*i*,*i*+1_ between residues *i* and *i* + 1 and ***r***_*j*,*j*+1_ between residues *j* and *j* + 1. The distribution of the angle θ_*ij*_ between vectors ***r***_*i*,*i*+1_ and ***r***_*j*,*j*+1_ can be used to calculate the orientation correlation of the Cα‐Cα vectors. We consider the ensemble mean of cos θ_*ij*_,

(5) $$\left\langle \cos\theta_{\mathrm{ij}}\right\rangle =\sum_{c=1}^{C}w_{c}\cos\theta_{\mathrm{ij},c}$$

and its variance

(6) $$\sigma_{\mathrm{ij}}^{2}=\sum_{c=1}^{C}w_{c}{\left(\cos\theta_{\mathrm{ij},c}-\left\langle \cos\theta_{\mathrm{ij}}\right\rangle \right)}^{2}$$

For a large *C* and a uniform distribution of θ_*ij*_, $\sigma_{\mathrm{ij}}^{2}$ approaches ½. If θ_*ij*_ is the same for all conformers, $\sigma_{\mathrm{ij}}^{2}$ is zero. Hence, the site‐pair correlation parameter $K_{\mathrm{ij}}=1-\sqrt{2}\sigma_{\mathrm{ij}}$ takes values from 0 for no correlation to 1 for perfect correlation. To characterize the order at residue *i* in the context of the entire chain, we sum over all *j* and obtain the site‐specific order parameter

(7) $$o_{i}=\frac{1}{N}\sum_{j=1}^{N}K_{\mathrm{ij}.}$$

By considering the correlation of Cα‐Cα vectors over the entire chain, *o*_*i*_ abstracts from local chain flexibility. The parameter is still site‐specific, as orientation correlations in IDRs and IDPs decrease with increasing sequence distance |*i* − *j*|.

To obtain a disorder parameter that is sensitive to local flexibility, we consider the circular variance of the Ramachandran angles ϕ_*i*_ and ψ_*i*_ (MacArthur & Thornton, 1993). The circular variance of ϕ_*i*_ is given by

(8) $$R^{2}\left(\phi_{i}\right)={\left(\sum_{c=1}^{C}w_{c}\cos\phi_{i,c}\right)}^{2}+{\left(\sum_{c=1}^{C}w_{c}\sin\phi_{i,c}\right)}^{2},$$

where an analogous expression applies for $R^{2}\left(\psi_{i}\right)$. If all conformers have the same dihedral angles at residue *i*, each of the two circular variances is equal to one. For a large *C* and with a uniform distribution of dihedral angles, the circular variance tends to zero. Therefore, we can define a site‐specific flexibility parameter

(9) $$f_{i}=1-\frac{1}{2}R\left(\phi_{i}\right)-\frac{1}{2}R\left(\psi_{i}\right)$$

which ranges from 0 for identical dihedral angles for all conformers at the residue *i* to 1 for a uniform distribution of dihedral angles at the residue *i*.

### 2.3 Deviation from random‐coil behavior

In the limiting case of complete disorder, the conformer ensemble of IDPs and IDRs can be modeled by an analytical Flory random coil (Alston et al., 2023; Ritsch et al., 2021). This concept can also be applied to sections of the chain. In the following, we extend such a concept that was originally implemented in the predecessor program MMM (Jeschke, 2021). For this purpose, we consider root mean square end‐to‐end distances for sections between residues *i* and *j* with sequence length Δ*N* = *j* − *i* (*j* > *i*). A chain of length *N* represented by a Cα trace contains *N* − Δ*N* segments of sequence length Δ*N*. Since Δ*N* ranges from 1 to *N* − 1, the ensemble can be represented by *N*(*N*‐1)/2 root mean square end‐to‐end distances of the chain sections, which are defined as follows:

(10) $$r_{\mathrm{ee},i,j}=\sqrt{\sum_{c=1}^{C}w_{c}r_{i,j,c}^{2}}$$

If the entire chain behaves like a Flory random coil with uniform solvent quality along the sequence, we expect *r*_ee,*i*,*j*_ to scale with Δ*N* = *j* − *i* as follows

(11) $$r_{\mathrm{ee}}\left(\Delta N\right)=b_{0}\;{\Delta N}^{\nu }$$

Here, *b*_0_ is the Kuhn length, which we treat as a fitting parameter. We set the Kuhn length to be the same for all sections of the same chain. In the long chain limit of a homopolymer, the scaling exponent ν can take values of 1/3 in a poor solvent, of ½ in a θ‐solvent that exactly balances the exclusion–volume interactions of the chain, and of 0.588 in a good solvent. Intermediate values may occur for IDPs and IDRs, which are heteropolymers of moderate chain length. While denatured proteins tend toward the good solvent case, careful evaluation of experimental data from SAXS and single‐molecule FRET studies has shown that water or buffer solutions are θ‐solvents for many IDPs (Best, 2020). Highly charged IDPs are an exception. They tend toward the good solvent case or may even have scaling exponents >0.588.

Since IDPs and IDRs are heteropolymers, the solvent conditions can vary along the chain. In addition, the tendency to form secondary structure elements can lead to deviations from random coil behavior. Therefore, *r*_ee,*i*,*j*_ is not necessarily the same for all chain sections with the same sequence length Δ*N*. We define the average of *r*_ee_ over all sections with the same sequence length,

(12) $${\bar{r}}_{\mathrm{ee}}\left(\Delta N\right)=\frac{\sum_{j-i=\Delta N}r_{\mathrm{ee},i,j}}{N-\Delta N}$$

**FIGURE 1:**
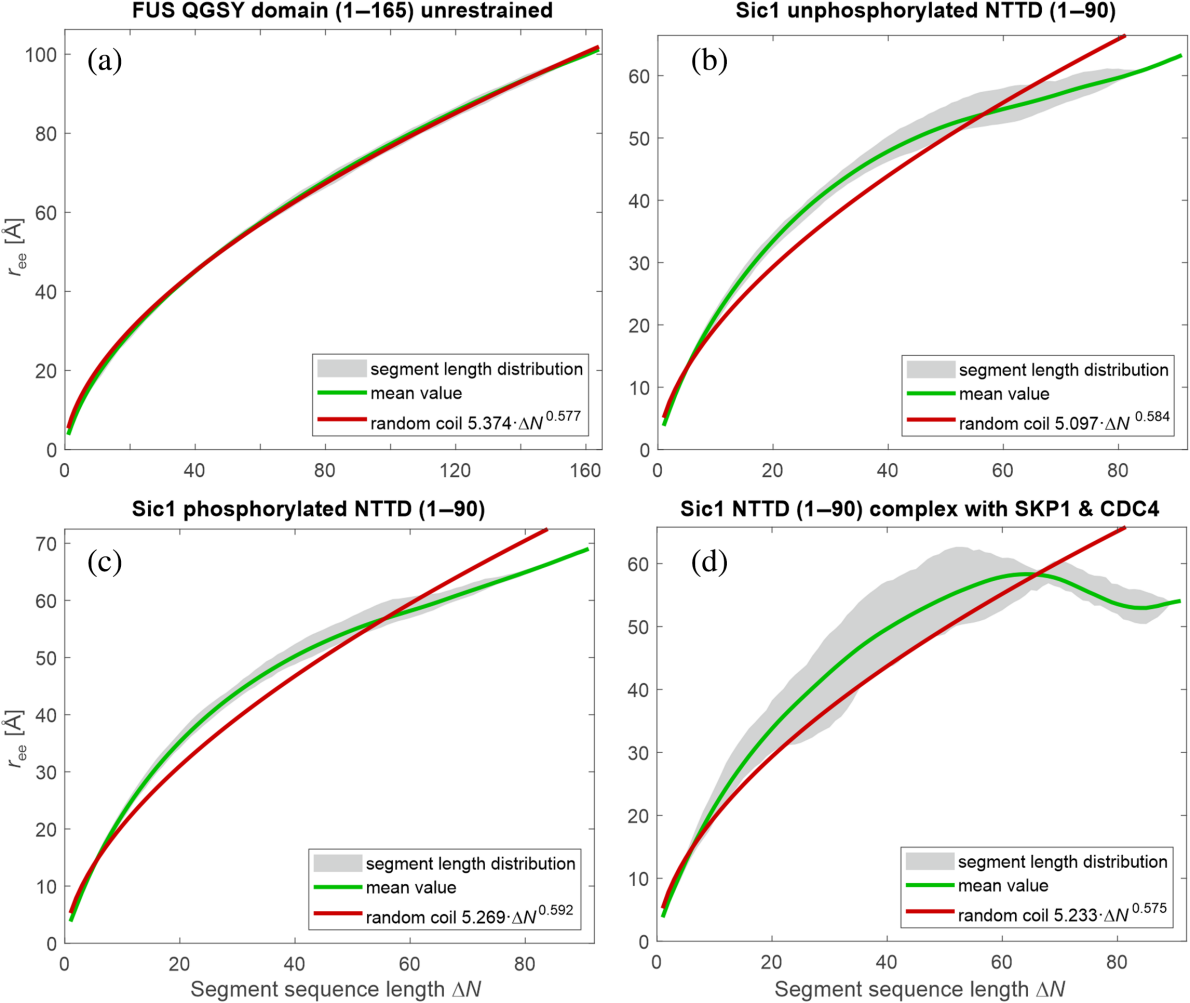
Plot of the root mean square end‐to‐end distances *r*_ee,*i*,*j*_ of all chain sections with sequence length Δ*N* (gray area) vs. Δ*N* for (a) unrestrained fused in sarcoma QGSY‐rich domain and (b) unphosphorylated Sic1 N‐terminal targeting domain (NTTD) (PED00159; Gomes et al., 2020) (c) phosphorylated Sic1 NTTD (PED00161; Gomes et al., 2020) (d) phosphorylated Sic1 NTTD in complex with SKP1 and CDC4 (combined ensembles of PED00014; Mittag et al., 2010). The green line denotes the mean values for given Δ*N*, while the red line represents a fit to a scaling law expected for random coils. The fitting parameters are given in the inset.

If the chain deviates from Flory's random coil behavior, ${\bar{r}}_{\mathrm{ee}}\left(\Delta N\right)$ may not obey Equation (11). We illustrate such deviation by plotting all *r*_ee,*i*,*j*_ against Δ*N* (gray area in Figure 1) along with a fit of Equation (11) to all *r*_ee,*i*,*j*_ (red line) and the dependence of ${\bar{r}}_{\mathrm{ee}}$ on Δ*N* (green line). For an unconstrained ensemble of the QGSY‐rich domain of the fused in sarcoma (FUS) protein, we find a nearly perfect Flory random coil behavior with a scaling exponent close to the conditions for good solvents (Figure 1a). This ensemble, generated with the Flex module of MMMx (Jeschke, 2016), is based on residue‐specific Ramachandran diagrams derived from random coil sections in PDB structures (Hovmöller et al., 2002). In contrast, the experimentally informed ensembles of the N‐terminal targeting domain of Sic1 (Gomes et al., 2020; Mittag et al., 2010) deviate from Flory random coil behavior (Figure 1b–d). For this protein, the *r*_ee,*i*,*j*_ for the same Δ*N* exhibit a substantial distribution, and the dependence of ${\bar{r}}_{\mathrm{ee}}$ on Δ*N* does not conform to Equation (11). Additional examples of other proteins are shown in Figure S1, where measles virus nucleoprotein 400–425 (Ozenne, Schneider, et al., 2012) and NUS 1313–1390 (Fuertes et al., 2017) are well approximated by Flory random coils with scaling exponents intermediate between the θ‐solvent and good solvent cases.

To illustrate the local expansion or compression of the chain, we resolve the data for the individual sections in a two‐dimensional plot. In the first approach, which we previously introduced as proximity matrix ***P*** (Jeschke, 2021), we calculate and display the matrix elements

(13) $$P_{\mathrm{ij}}=\frac{r_{\mathrm{ee},i,j}-b_{0}\;{\left|j-i\right|}^{\nu }}{b_{0}\;{\left|j-i\right|}^{\nu }},$$

where *b*_0_ and ν are the best‐fit values for the Kuhn length and the scaling exponent, respectively. We obtain them by minimizing the mean square deviation of Equation (11) from all *r*_ee,*i*,*j*_. An analogous compactness matrix ***C*** with the elements *C*_*ij*_ can be defined with the gyration radius of the sections instead of the *r*_ee,*i*,*j*_ (Jeschke, 2021). A two‐dimensional scaling matrix with the elements *C*_*ij*_ + 1 has already been used for the analysis of integrative ensemble models of the protein Sic1 (Gomes et al., 2020).

For larger deviations from random‐coil behavior, the heterogeneous compaction and expansion are better represented by the section length deviation matrix ***S*** with the elements

(14) $$S_{\mathrm{ij}}=r_{\mathrm{ee},i,j}-{\bar{r}}_{\mathrm{ee}}\left(\left|j-i\right|\right).$$

**FIGURE 2:**
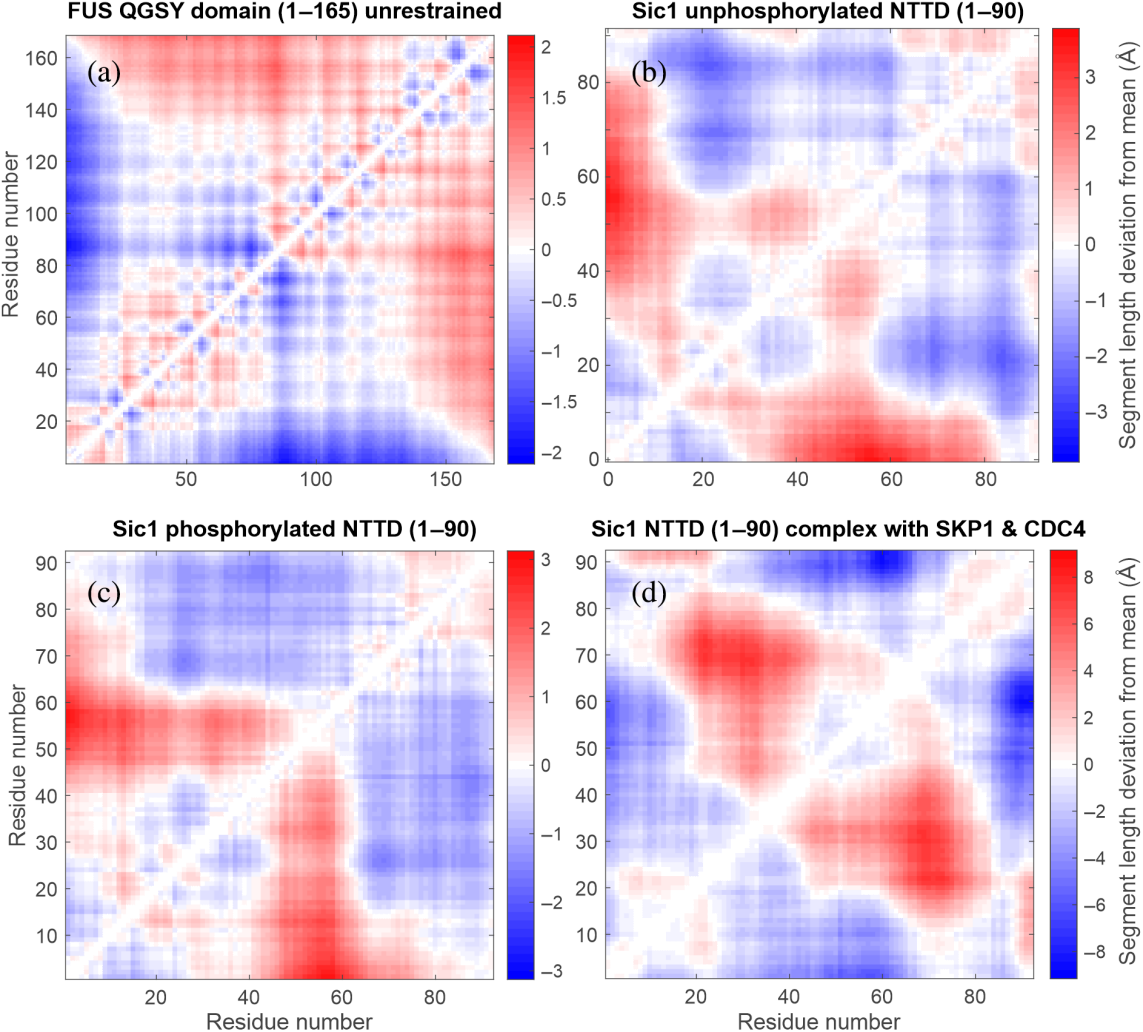
Deviation *S*_*ij*_ of section root mean square end‐to‐end distance from the average over sections with the same sequence length for (a) unrestrained fused in sarcoma (FUS) QGSY‐rich domain, (b) unphosphorylated Sic1 N‐terminal targeting domain (NTTD) (PED00159; Gomes et al., 2020) (c) phosphorylated Sic1 NTTD (PED00161; Gomes et al., 2020), and (d) phosphorylated Sic1 NTTD in complex with SKP1 and CDC4 (combined ensembles of PED00014; Mittag et al., 2010). Blue regions correspond to sections that are more compact than other sections of the same sequence length and red regions to sections that are more expanded.

This is illustrated in Figure 2, where red regions represent extended sections and blue regions represent compacted sections. Local expansion and compaction by up to 2 Å is observed even in the unconstrained ensemble of the QGSY‐rich FUS domain, which closely resembles a Flory random coil in good solvent. In the experimental ensemble of unphosphorylated Sic 1 (PED00159), the sections between the N‐terminus and residues 45–65 are about 3 Å more elongated than the average section with the same sequence length (Figure 2b). The sections between residues 14–25 and the C‐terminus are more compact than the average sections of the same sequence length.

### 2.4 Domain partitioning and structure classes

The degree of disorder in a protein determines which approaches are suitable for experimental structure determination and modeling. Fully folded proteins and folded domains are best studied using experimental techniques that provide atomic resolution, such as x‐ray crystallography, cryo‐electron microscopy, or NMR spectroscopy. IDPs and IDRs are usually investigated using small angle scattering techniques, single‐molecule FRET, NMR, or EPR spectroscopy. Data from several of these techniques are often integrated to compensate for the shortcomings of the individual techniques. MD data can support ensemble modeling in hybrid approaches (Lindorff‐Larsen et al., 2004). Proteins containing both folded domains and IDRs generally require such integration of data from multiple techniques, especially when the relative arrangement of multiple folded domains is distributed (Dorn et al., 2023; Huang et al., 2014). Similarly, the presence of both folded domains and IDRs complicates modeling by MD approaches (Fu et al., 2019; Jeschke, 2022). For these reasons, we consider it convenient to classify proteins into fully folded (F, folded), single folded domains with terminal IDRs (Sf), multiple folded domains with connecting IDRs (Mf), and IDPs (D).

Such classification requires the partitioning of a protein into folded domains and IDRs, which also enables the design of experiments for structure determination. Tools exist for predicting disordered regions of a peptide sequence with reasonably high confidence (Conte et al., 2023). Predictions for the human proteome have shown that the predicted local distance difference test (pLDDT) score of AF2 is a competitive disorder predictor and that AF2 can provide accurate folded domain structures in most cases (Ruff & Pappu, 2021; Tunyasuvunakool et al., 2021). A recent critical evaluation of intrinsic disorder prediction of proteins showed that pLDDT works well for the linker dataset, which is particularly relevant for the task at hand. In contrast, the pLDDT score did not perform well for the binder dataset (Conte et al., 2023). In the following, we use SETH (Ilzhöfer et al., 2022) as a predictor that performs well for both datasets and eSpritz (Walsh et al., 2012) as a predictor that performs at a reasonable level and is sufficiently fast for processing the entire human proteome.

In addition, AF2 provides a PAE matrix in which folded domains correspond to square submatrices with low PAE. Recent work has shown that PAE correlates with the standard deviation of Cα distances observed in MD simulations and that the PAE can be used to construct an elastic network model (ENM; Jussupow & Kaila, 2023). We therefore hypothesized that the PAE matrix would provide a more robust domain partitioning than a one‐dimensional disorder prediction along the chain. AF2 structure predictions are based on the assumption that coevolution implies interaction between amino acids. Therefore, PAE values between domains can even indicate whether folded domains interact strongly, weakly, or not at all. Such information is also useful for experimental design. However, it should be noted that coevolution can also take into account the interaction of a protein with binding partners, so the PAE matrix is not necessarily related to the structure of the free protein.

For the domain partitioning algorithm of MMMx, we assume a minimum size of 25 residues for a folded domain, which is justified by an analysis of the domain size distribution in the protein database (Jones et al., 1998). We classify an end segment as an IDR if it contains at least 10 residues that do not belong to a folded domain. For the assignment of folded domains, we search for square submatrices with a mean PAE of <10 Å (see Section 4). For comparison, we attempted to perform domain partitioning by analyzing one‐dimensional disorder predictions, where we consider a contiguous section of at least 25 ordered residues as a folded domain.

**FIGURE 3:**
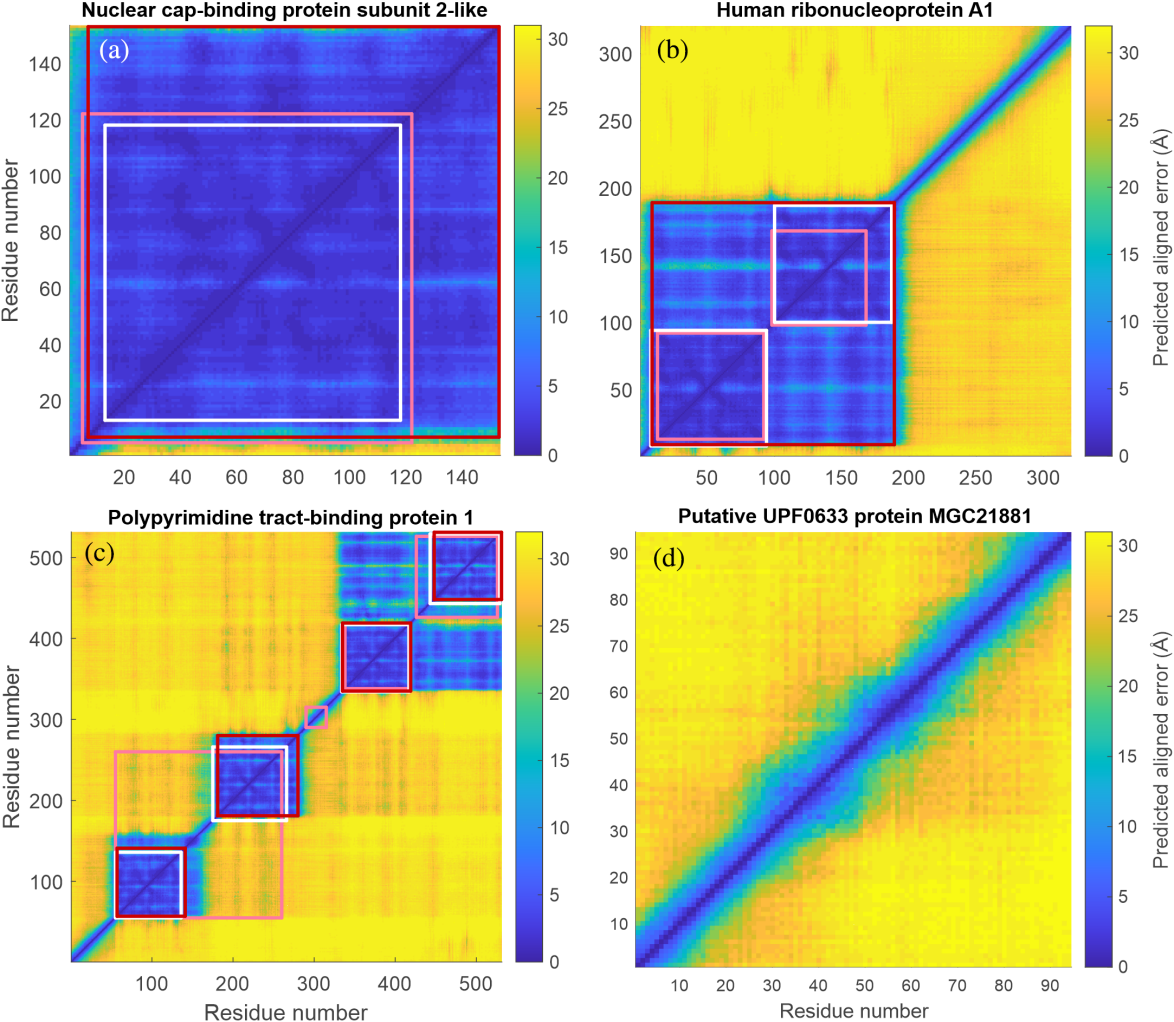
Domain partitioning and classification of proteins by MMMx based on the predicted aligned error output by AlphaFold2. Detected domains are marked by red squares. For comparison, the domain partitioning based on the perturbation predictions of SETH (Ilzhöfer et al., 2022, white squares) and eSpritz (Walsh et al., 2012, pink squares) is shown. (a) Nuclear cap‐binding protein subunit‐2 like (UniProt A6PVI3) is recognized as completely folded (class F). (b) Human ribonucleoprotein A1 (UniProt Q6IPF2) is recognized as having a single folded domain and a long C‐terminal IDR (class Sf). (c) Polypyrimidine tract‐binding protein 1 (UniProt P26599) is recognized as having four folded domains joined by flexible IDRs and a long N‐terminal IDR (class Mf). (d) Putative UPF0633 protein MGC21881 (UniProt A6NN06) is recognized as fully disordered (class D).

Figure 3 shows the AF2‐PAE matrices and domain assignments for examples from each of the four classes. Nuclear cap‐binding protein subunit‐2 like (UniProt ID A6PVI3, Figure 3a) is assigned to class F based on the PAE matrix, in agreement with the model of this protein in the SwissModel database. In contrast, SETH (Ilzhöfer et al., 2022) assigns IDRs of more than 10 residues at both the N‐ and C‐termini, and eSpritz (Walsh et al., 2012) assigns a C‐terminal IDR. Human ribonucleoprotein A1 (UniProt ID Q6IPF2, Figure 3b) is assigned to class Sf based on the PAE matrix, with a single folded domain and a C‐terminal IDR. This is in agreement with various x‐ray and NMR structures known for the folded domain. Both SETH and eSpritz recognize the two RNA recognition motifs (RRMs) of this protein as separate folded domains. All disorder predictors correctly assign (Dorn et al., 2023) polypyrimidine tract‐binding protein 1 (UniProt ID P26599, Figure 3c) to class Mf. PAE and SETH correctly recognize four RRMs, although the two approaches differ in the detailed assignment of these domains. In contrast, eSpritz does not recognize the IDR linker between RRM1 and RRM2. None of the approaches predicts that RRM3 and RRM4 move as a single rigid body, as found experimentally (Dorn et al., 2023; Gmeiner et al., 2017; Vitali et al., 2006). However, a visual inspection of the PAE matrix is consistent with this experimental result. All four approaches assign the putative UPF0633 protein MGC21881 (UniProt ID A6NN06, Figure 3d) as an IDP. Further examples are shown in Figure S2. In general, we find that domain assignment based on eSpritz is less reliable than based on the other two approaches. The AF2 PAE‐based approach is faster than the SETH‐based approach because the PAE matrices are precomputed and accessible from a database.

### 2.5 Distribution of structure classes over the human proteome

Analysis of AF2 predictions for the human proteome has shown that IDRs and IDPs play an important role that has not yet been reflected in the treatment of intrinsic disorder in molecular and structural biology research (Ruff & Pappu, 2021). For the development of methods aimed at overcoming this lack of information on IDRs and IDPs, it is of interest to determine how the human proteome is distributed across structural classes. To this end, we analyzed the set of AF2 predictions for the human proteome in the AlphaFold Protein Structure Database (AF‐PSD, 95% coverage; Tunyasuvunakool et al., 2021). As a single‐valued disorder parameter, we calculated mean PAE for 20,195 human proteins with at least 50 residues. For this purpose, we neglected residue pairs with a sequence distance |*j* − *i*| < 10, where the PAE is also reduced in IDPs. We find that this mean PAE is broadly distributed between 3 and 29 Å, with most proteins having a mean PAE >10 Å (Figure S3). Considering that a PAE of 3 Å indicates a well‐defined folding of the entire chain and that the PAE is capped at 31.75 Å, this result confirms earlier assessments that human proteins cover the entire continuum of order and disorder. The PAE‐based algorithm assigns only (4.2% ± 1.3%) of all proteins to class F (fully folded) and (59.9% ± 3.2%) to class Mf with multiple domains connected by IDRs (Table 1). The latter result could be of great significance, as very few ensemble structures have been published within the Mf class to date. There are also very few experimental ensemble structures for class Sf, which contributes another (27.3% ± 3.3%). Performing the same analysis with an eSpritz‐based domain partitioning reveals a much smaller proportion of IDPs and a much larger proportion of fully folded proteins. However, this algorithm also predicts that the Sf and Mf classes account for about two thirds of all human proteins. Given the results shown in Figures 3 and S2, we believe that the PAE classification is more reliable. The differences could be due to the fact that the detection of contiguous folded domains by a measure for residue pairs is more robust than by a measure for individual residues, or that domain detection is very sensitive to the accuracy of predicting disorder at individual residues. The latter reason is suggested by the better agreement between the SETH‐ and PAE‐based approaches, as shown by the anecdotal examples in Figures 3 and S2. Separating the two possible reasons would require a large‐scale study with computationally intensive SETH predictions, which is beyond the scope of this article.

**TABLE 1:** Distribution of 20,195 human proteins among the order/disorder classes based on AlphaFold2 (AF2)‐predicted aligned error (PAE) and eSpritz disorder prediction.

Approach	F	Sf	Mf	D
AF2 PAE	4.2% ± 1.3%	27.3% ± 3.3%	59.9% ± 3.2%	8.6% ± 1.4%
eSpritz	31.5% ± 0.9%	23.0% ± 0.2%	44.0% ± 0.7%	1.5% ± 0.1%

*Note*: F, fully folded; Sf single folded domain with at least one terminal intrinsically disordered region (IDR) of at least 10 residues, Mf multiple folded domains connected by IDRs, D, disordered (intrinsically disordered protein). See Section 4.1 for the explanation of the uncertainty estimates.

### 2.6 Experiment design

We model IDRs in proteins of classes MF and Sf and IDPs in class D by a Monte Carlo approach based on residue‐specific Ramachandran angular distributions (Hovmöller et al., 2002). This approach, implemented in the Flex module of MMMx (Jeschke, 2021) based on a previously developed algorithm (Jeschke, 2016), accounts for experimental distance distribution restraints to improve the efficiency of sampling the conformer space. As an extension of the previous algorithm, the Flex module enables the consideration of non‐Gaussian distance distribution restraints by applying von Neumann rejection sampling with a predefined acceptance fraction. To characterize structural changes of folded domains upon binding of ions, small‐molecule ligands, other proteins, or nucleic acids, MMMx relies on the deformation of an ENM by forces calculated from distance restraints (Zheng & Brooks, 2006). For proteins in class Mf, we model ensemble structures using the RigiFlex approach, which can specify both the distributed relative arrangement of folded domains and the conformational ensemble of IDR linkers and terminal sections (Jeschke, 2021; Jeschke & Esteban‐Hofer, 2022). All of these approaches rely on distance distributions between spin labels. In the following, we discuss the selection of suitable spin labeling sites from the PAE matrix and AF2 structure prediction.

The ExperimentDesign module of MMMx uses modeling of the conformational distribution of spin labels by a rotamer library (Polyhach et al., 2011; Polyhach & Jeschke, 2010), to find suitable spin label sites and site pairs. For this purpose, we import a single structure or an ensemble structure from the PDB, the PED, the AF‐PSD, Zenodo, or a local file. With the exception of automatically creating a RigiFlex script template from an AF2 prediction (see below), we recommend starting with a scan of the spin label sites (Polyhach & Jeschke, 2010), followed by manual removal of sites from the list that are known disease mutants or interaction sites.

ENM modeling uses distance restraints to generate forces that act on a bead and spring model of a protein's Cα trace (Jeschke, 2012b; Jeschke, 2012c; Zheng & Brooks, 2006). The MMMx implementation extends the previous implementation by ensemble modeling based on distance distributions sampled with a Monte Carlo approach. By considering the normal modes of an anisotropic ENM, the optimal pairs of sites for measuring distance distributions can be derived. Given a list of *L* potential spin‐label sites, the algorithm considers the lowest *L*(*L* − 1)/2 normal modes of the ENM. Starting from the mode with the lowest energy, it selects the pair for each mode that experiences the largest distance change when the mode is activated.

When designing experiments for RigiFlex we first perform domain partitioning based on an AF2 prediction (see above) to identify folded domains and IDRs. Then, we write a rigid body template file in PDB format containing only the folded domains. For each rigid body, we select three reference sites for spin labeling. For this purpose, we perform a site scan for the folded domain. We calculate the area of the triangle between the mean spin label positions for each triple of potential labeling sites and select the triple with the largest area. The module writes a RigiFlex template script with a Rigi section to model the distributed arrangement of the folded domains and Flex sections for each IDR.

We advise users to check whether experimental structures exist for the rigid bodies and whether proposed reference sites are known disease mutants or interaction sites. For the prediction of reference sites from an edited site scan list, we have implemented the keyword RBreference.

**FIGURE 4:**
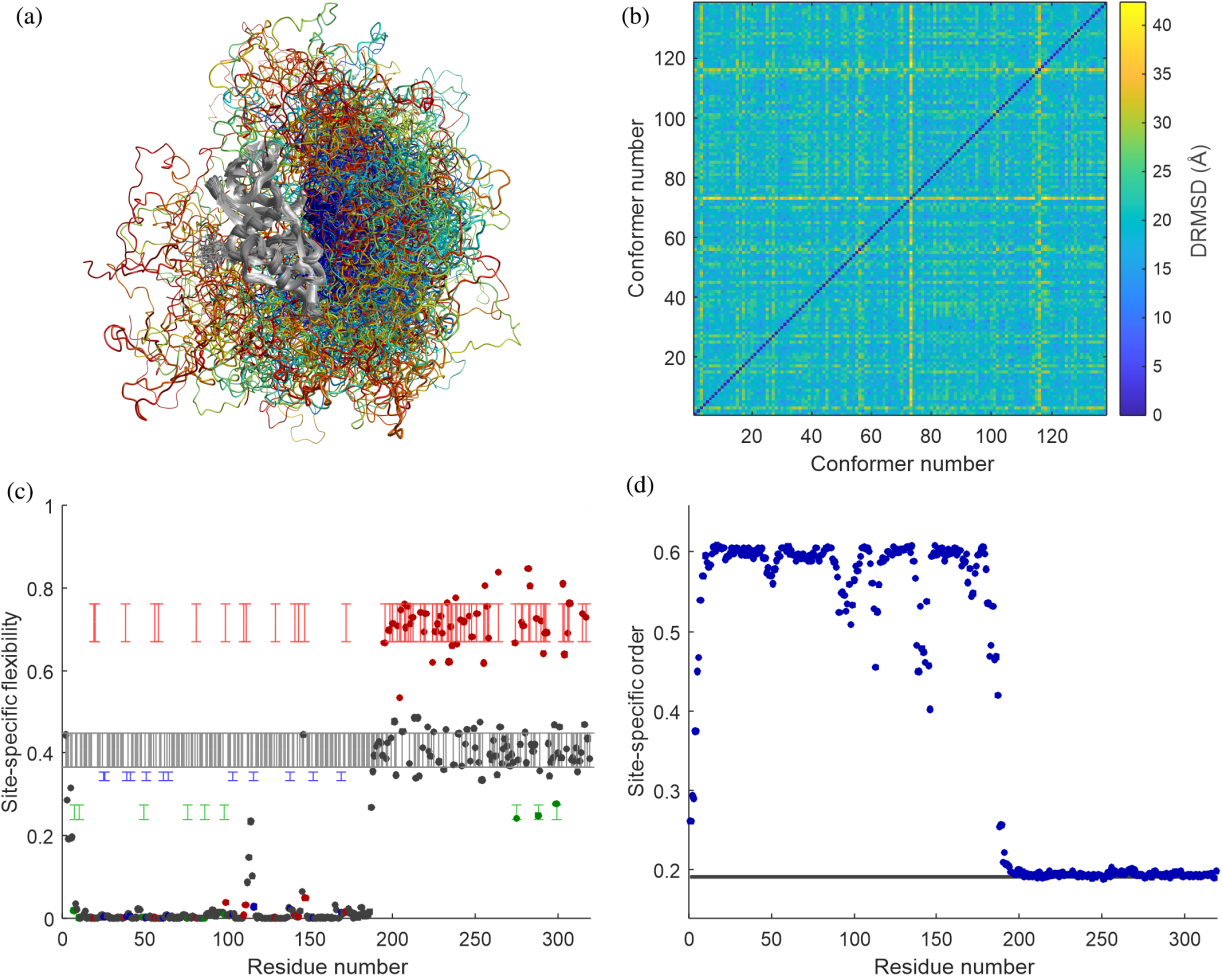
RigiFlex ensemble of hnRNP A1 and its analysis. (a) Snake visualization of the ensemble with MMMx/MMM. The ensemble is superimposed on the two folded RRMs (residues 1–187, gray ribbon model). The IDR is visualized by coils with a color gradient from blue (N‐terminal residue 188) to red (C‐terminal residue 321), whereas coil radius encodes weight of the conformers. (b) Two‐dimensional plot of distance root mean square deviation (DRMSD) that visualizes pairwise distance of conformers in abstract conformer space. (c) Plot of site‐specific flexibility color coded for Gly (red), Pro (green), Thr (blue), and all other residues (black). The ranges indicated by vertical bars correspond to the random‐coil expectation. (d) Plot of site‐specific order (blue circles). The gray range corresponds to the random‐coil expectation.

Two ensemble models generated using the MMMx approach are shown in Figures 4, S4, and S5. For the ensemble of hnRNP A1 (Ritsch et al., 2022), residues 1–187 were treated as rigid bodies, except that the rigid body structure was varied between 20 conformers from the NMR ensemble with PDB identifier 2lyv. The C‐terminal IDR (residues 188–321) was generated using Flex and integrative ensemble reweighting with DEER and SAXS restraints was performed using the EnsembleFit module. Figure 4a shows a snake visualization generated with MMMx and MMM. A plot of the pairwise DRMS (Figure 4b) indicates a fairly homogeneous distribution across the conformer space, except for conformers 73 and 116, which are distant from most other conformers. Figure 4c shows that some residues in the IDR are more flexible and other residues less flexible than expected from Ramachandran statistics, while Figure 4c shows that the IDR is as disordered as a random coil, although the chain extension is heterogeneous, as discussed previously (Ritsch et al., 2021). For PTBP1 in complex with EMCV‐IRES D‐F (Figure S4), only ensemble reweighting with DEER, SAXS, and SANS restraints was performed with MMMx (Dorn et al., 2023). Analysis of this ensemble reveals a large aligned uncertainty between the folded domains (Figure S5a), a division of the ensemble into two subensembles by DRMS (Figure S5b), a very high flexibility of the IDR connecting RRM2 and RRM3 (Figure S5c), and the absence of significant site‐specific ordering in both interdomain linkers (Figure S5d). Further ensembles generated with MMMx can be found in the PED with identifiers PED00493, PED00494, and PED00495 (FUS NTD in different environments) and with identifier PED00496 (tandem RRMs of SRSF1 joined by a flexible linker).

### 2.7 Analysis of site‐specific order and flexibility

**FIGURE 5:**
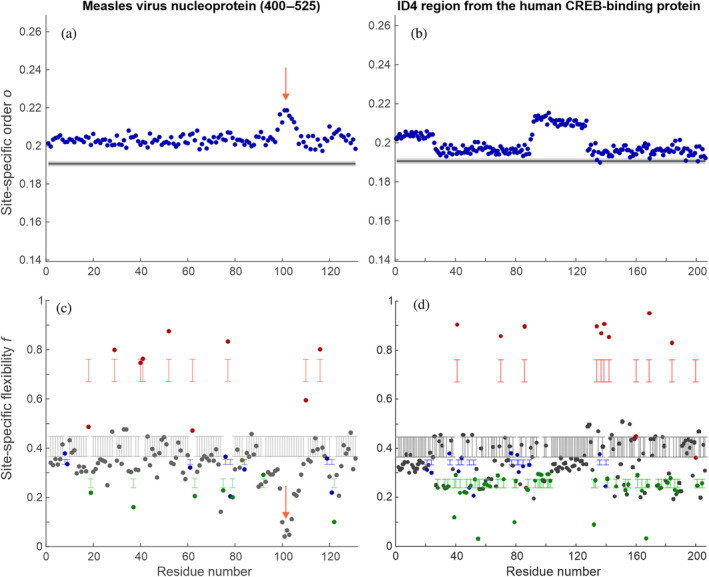
Analysis of local order (a,b) and Ramachandran flexibility (c,d) on ensembles of (a,c) measles virus nucleoprotein (residues 400–525, MeV‐1, PED00020, ensemble e001; Ozenne, Schneider, et al., 2012) and (b,d) the ID4 region from the human CREB‐binding protein (CBP‐ID4, PED00216; Piai et al., 2016). The orange arrows in (a) and (c) denote a short rigidified section in MeV‐1 (residues 99–104) that is revealed by this analysis. The gray lines in (a,b) denote the expectation for a random coil based on analysis of an unrestrained ensemble of the N‐terminal domain of fused in sarcoma (FUS N‐terminal domain [NTD], residues 1–267, see Figure S6). The colored ranges in (c,d) denote the expectations for a random coil for Gly (red), Pro (green), Thr (blue), and all other residues (black) based on the same analysis of FUS NTD.

We validated the site‐specific measures of disorder presented in Section 2.2 on an ensemble of the N‐terminal domain (NTD, residues 1–267) of the protein FUS with *C* = 5000. This ensemble is based only on the residue‐specific Ramachandran angular distribution for the loop region (Hovmöller et al., 2002). We find a mean site‐specific disorder parameter of 0.1906 with a standard deviation of only 0.0007 (Figure S6). For this case of a nearly perfect random coil, the variation of the site‐specific order parameter *o*_*i*_ along the chain is negligible. In contrast, analysis of an ensemble of the very weakly ordered nucleoprotein of measles virus (residues 400–525; Ozenne, Schneider, et al., 2012, Figure 5a) shows a short stiffened segment near residue 100 (orange arrow). For the ID4 region of the human CREB‐binding protein (CBP‐ID4, PED00216), the same analysis recognizes the four different segments discussed by Piai et al (Piai et al., 2016). with slightly less order assigned to segments rich in Pro but also in Gly residues (Figure 5b).

The site‐specific order parameter *o*_*i*_ distinguishes well between folded domains and IDRs and is sensitive to a rather weak order. On the other hand, it masks the differences in the inherent flexibility of the different residues, with Gly likely to be the most flexible and Pro the least flexible. For the unrestrained ensemble of FUS NTD, we find that the site‐specific flexibility *f*_*i*_ indeed differs between Gly, Pro, and the other residues (Figure S6). Surprisingly, we also find a slight but significant difference between Thr and the other residue types. In figure 9 of the paper by Hovmöller et al. (2002), from which we extracted the residue‐specific Ramachandran angular distributions, only regions in the second and third quadrants are populated for Thr. The population in the third quadrant is close to the boundary of the second quadrant. Other residue types, with the exception of Ile and Pro, also have some population in the first quadrant. The population for Ile is somewhat more widely distributed in the second and third quadrants than for Thr. Since the deviation of Thr from all residues other than Gly, Pro, and Thr is small, we believe that it can be explained by these features in the Ramachandran diagrams.

We take the site‐specific flexibilities *f*_*i*_ obtained for unrestrained FUS NTD as a reference for the random coil. Their mean values and standard deviations are listed in Table 2. Ramachandran flexibility plots in MMMx are color‐coded for these residue types and give 95% confidence intervals of the random coil values (Figure 5c,d). For MeV‐1 (Ozenne, Schneider, et al., 2012), such analysis again shows the short stiffened segment near residue 100 (orange arrow in Figure 5c), whereas for CBP‐ID4 (Piai et al., 2016) it shows that the most disordered segments contain some highly flexible Gly residues (Figure 5d).

**TABLE 2:** Mean values $\left\langle f_{i}\right\rangle$ and standard deviations $\sigma \left(f_{i}\right)$ for the site‐specific flexibility of different residue types in an unrestrained ensemble of the N‐terminal domain (residues 1–267) of the protein fused in sarcoma.

	Gly	Pro	Thr	All others
$\left\langle \left.f_{i}\right\rangle \right.$	0.7158	0.2574	0.3437	0.4065
$\sigma \left(f_{i}\right)$	0.0226	0.0089	0.0051	0.0203

### 2.8 Ensemble comparison

**FIGURE 6:**
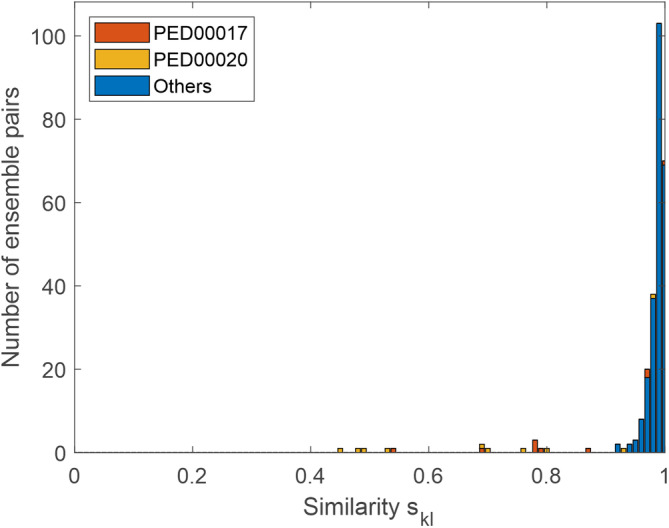
Histogram of 262 similarity measures *s*_*kl*_ defined in Equation (4). Pairs of ensembles in the Protein Ensemble Database (PED) that belong to the same state of the same protein are compared.

There are several approaches for comparing ensembles of folded proteins (Aina et al., 2023; Brüschweiler, 2003; Lindorff‐Larsen & Ferkinghoff‐Borg, 2009; Tiberti et al., 2015). Recently, the Wasserstein distance‐based tool WASCO has been introduced for site‐pair resolved comparison of ensembles of IDPs (González‐Delgado et al., 2023). Inter‐residue matrices of the median and standard deviation of the distance between residues can also reveal similarities and differences between ensembles of IDPs (Lazar et al., 2020). Based on a general analytical theory for heteropolymers (Huihui & Ghosh, 2020), it is possible to compare IDP ensembles with different sequences using inter‐residue distance profiles and the sequence charge decoration matrix (Huihui & Ghosh, 2021). The similarity measure *s*_*kl*_ defined in Section 2.1 enables fast comparison within groups of multiple ensembles by providing a single value for each ensemble pair. This measure can therefore complement the more sophisticated approaches. Here, we first validate the definition of *s*_*kl*_ by comparing ensembles for the same state of the same protein. To this end, we searched the PED for entries containing at least two ensembles. For efficiency reasons, we excluded entries with ensembles containing more than 1000 conformers. Among the total of 489 entries (Ghafouri et al., 2023), we found 11 multiensemble entries containing between 3 and 13 ensembles. For most pairs of ensembles of the same state of the same protein, *s*_*kl*_ is in the range between 0.95 and 1 (Figure 6). All *s*_*kl*_ below 0.9 belong to the two PED entries 17 and 20. We discuss the case with the lowest *s*_*kl*_ (ensembles 2 and 4 of entry 20) in the Supporting information S1 and find that the overall shape of the conformers is indeed different between the two ensembles, although they both agree well with the NMR restraints.

We then tested whether *s*_*kl*_ discriminates between ensembles of the same protein in different states. To do this, we used the 12 PED entries for the N‐terminal targeting domain of Sic1 corresponding to unphosphorylated Sic1, two different phosphorylated constructs, and the complex of one of these constructs with SKP1 and CDC4 (Table S1). Except for the complex, there are multiple PED entries for each state that differ in experimental data sets or modeling methodology. Three of the entries contain three ensembles each, while the other nine entries contain a single ensemble. We find a high similarity between the ensembles corresponding to the same state (Figure 6). The deviation of *s*_*kl*_ from 1 in these cases is most likely due to the small ensemble sizes (10–16 conformers). We also find a high similarity between the ensembles of free unphosphorylated and free phosphorylated Sic1. This is consistent with the very similar deviations from random coil behavior and the similar deviation matrices ***S*** shown for a pair of ensembles in Figures 1b,c and 2b,c.

**FIGURE 7:**
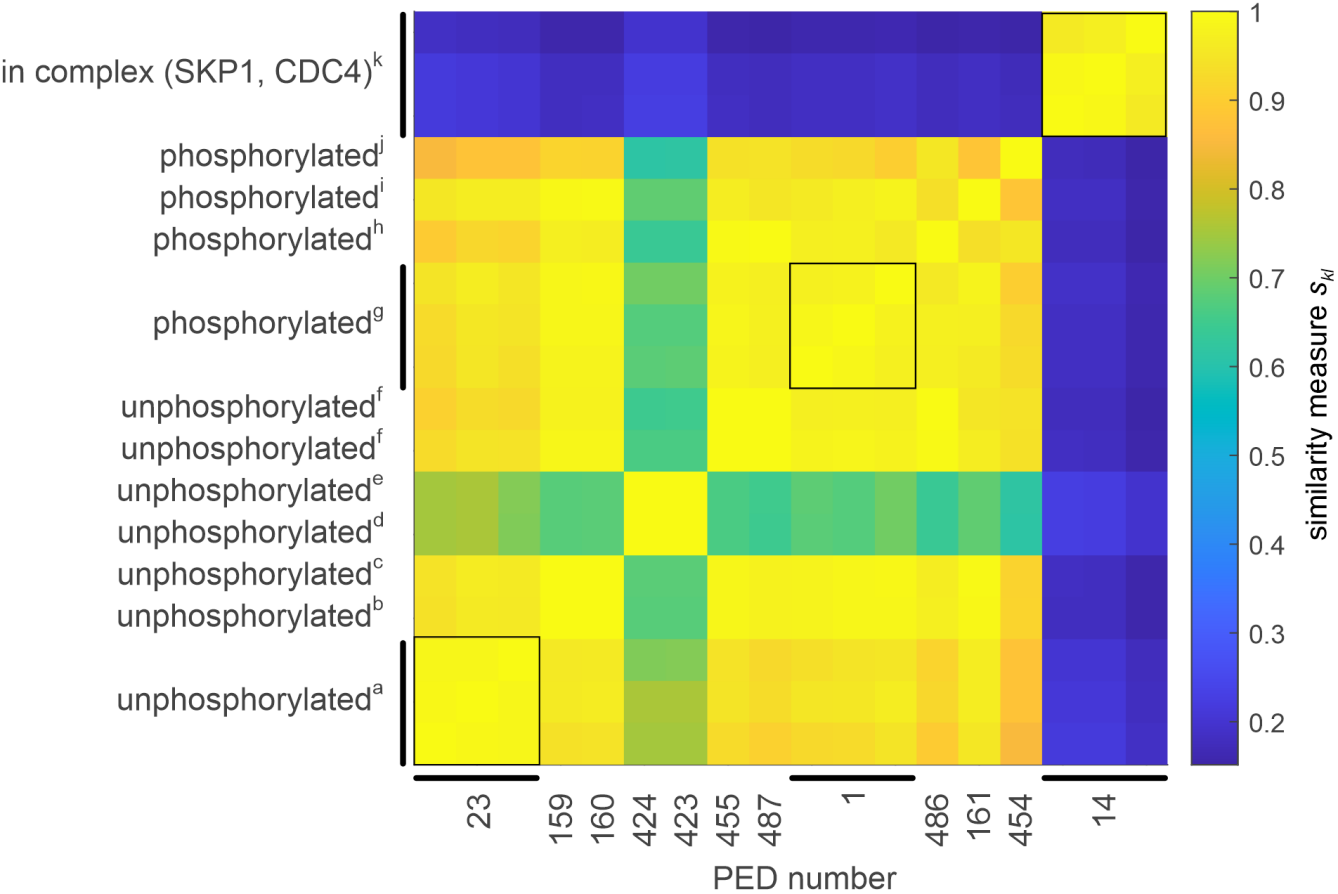
Similarity of the ensembles of Sic1 N‐terminal targeting domain for different states of the protein and for different experimental and modeling approaches. Specific details of the approaches can be found in the PED. Restraint sets include NMR residual dipolar couplings (RDC), chemical shifts (CS), paramagnetic relaxation enhancements (PRE), small‐angle x‐ray scattering (SAXS), and single‐molecule Förster resonance energy transfer (smFRET) data. Black squares indicate entries containing three ensembles. (a) NMR (RDC, CS, PRE) and SAXS, TraDES/ENSEMBLE; (b) NMR (PRE), SAXS, and smFRET, ENSEMBLE; (c) NMR (CS, PRE), SAXS, and smFRET, ENSEMBLE; (d) NMR(CS), IDPConformerGenerator; (e) no experimental data, IDPConformerGenerator; (f) no experimental data, idpGAN; (g) phosphorylated at sites 5, 33, 45, 69, 76, 80, NMR (RDC, CS, PRE) and SAXS, TraDES, ENSEMBLE; (h) phosphorylated at sites 5, 33, 45, 69, 76, 80, no experimental data, idpGAN; (i) phosphorylated at sites 2, 5, 33, 45, 69, 76, 80, NMR (PRE), SAXS, and smFRET, ENSEMBLE; (j) phosphorylated at sites 2, 5, 33, 45, 69, 76, 80, no experimental data, idpGAN; (k) phosphorylated at sites 5, 33, 45, 69, 76, 80, in complex with SPK1 and CDC4, NMR (RDC, CS, PRE) and SAXS, TraDES, ENSEMBLE.

Surprisingly, ensembles generated for the same state with different modeling approaches show a greater dissimilarity than ensembles generated with the same modeling approach for different phosphorylation states (Figure 7). This is particularly evident in the ensembles generated with IDPConformerGenerator (Teixeira et al., 2022) for unphosphorylated Sic1. These ensembles differ considerably from all other ensembles for this state. On the other hand, the ensembles generated with IDPConformerGenerator without experimental constraints and with chemical shift constraints are extremely similar (*s*_*kl*_ = 0.9996).

We find large differences between the three ensembles of the complex of phosphorylated Sic1 with SKP1 and CDC4 on the one hand and all ensembles of free Sic1 on the other hand. This is consistent with the different deviations from random coil behavior (Figure 1b–d) and the different deviation matrices ***S***. In particular, the deviations in section extension are much larger for the complex (Figure 2d) and the distribution of compacted and expanded sections across the chain is different. As another example of recognizing different ensembles, we present the case of the ensembles for the C‐terminal IDR of hnRNP A1 that we obtained earlier (Ritsch et al., 2021, 2022) with different sets of restraints and without restraints (Figure S7). In this case, all experimentally informed ensembles differ significantly from the ensemble obtained without restraints. The experimentally informed ensembles are very similar to each other, except for the ensemble informed by NMR‐PRE restraints only.

Visualization of differences between IDP ensembles is difficult when the two states have similar radii of gyration. Proteins of classes F and Sf can be superimposed on their folded domain by minimizing the mean square deviation of the coordinates. The same is also possible with respect to the individual folded domains in class Mf, although the choice of superimposed domain can lead to different clarity of representation (Figure S4). For the IDPs of class D, the root mean square deviation of the coordinates is not a suitable criterion for the relative orientation of the conformers. Instead, we superimpose the conformers by transforming them into the principal axes system (PAS) of their inertia tensor (see Section 4). This superposition leads to a representation that is as compact as possible. We represent the superimposed ensemble as a pseudoelectron density and provide coloring schemes for electrostatic interaction, cation–π interaction, and hydrophobic interaction.

**FIGURE 8:**
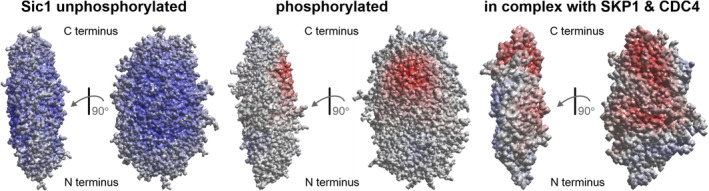
Comparison of the ensembles of the N‐terminal targeting domain of Sic1 for different states of the protein. The conformers were transformed into the principal axes system of their respective inertia tensor. The *y*‐axis (mean eigenvalue) is always upright in the paper plane. The viewing direction is either along *x* (left for each state) or along *z* (right). Shown are isosurfaces of pseudoelectron densities with a coloring corresponding to the mean positive (blue) or negative (red) charge density. The ensembles are from Protein Ensemble Database (PED) entries PED00159 (unphosphorylated; Gomes et al., 2020), PED00161 (phosphorylated; Gomes et al., 2020), and the three combined ensembles of PED entry PED00014 (in complex with SKP1 and CDC4; Mittag et al., 2010).

As an example, we compare ensembles for different states of Sic1 with the coloring scheme for electrostatic interactions (Figure 8). After phosphorylation, the shape of the ensemble hardly changes. Most of the surface of the ensemble representation is almost charge neutral in the phosphorylated state, while it is positive in the unphosphorylated state. However, there is a small part of the surface that has a negative charge density in the phosphorylated state. In the complex with SKP1 and CDC4 (Mittag et al., 2010), the charge density distribution changes, exposing the negative charge density on a larger part of the surface at the expense of the weak positive charge density in other parts. The visualization also shows a slight change in the shape of the ensemble in the complex. However, with only 44 conformers in the ensemble of the complex, the shape is more uncertain than in the other two ensembles, each comprising 500 conformers.

## 3 CONCLUSION

We have introduced new and improved features of the ensemble modeling and analysis package MMMx. The classification of proteins from the human proteome shows that a large proportion of these proteins contain multiple folded domains connected by IDR linkers. Together with proteins with a single folded domain and terminal IDRs longer than 10 residues, such heterogeneously disordered proteins make up about two thirds of the human proteome according to AF2 predictions. These structural classes are extremely underrepresented among the currently available ensemble models. In addition to the ensemble models of hnRNP A1 (Ritsch et al., 2021, 2022) and PTBP1/EMCV‐IRES D‐F (Dorn et al., 2023), the ensemble structure of tandem RRMs of SRSF1 connected by an IDR linker (PED00497) was also generated by the RigiFlex approach implemented in MMM (Jeschke, 2021) and MMMx (Jeschke & Esteban‐Hofer, 2022). Here, we have presented features of MMMx that are useful in planning such experimental studies. Ensemble models of proteins that have both folded domains and IDRs can also be generated without experimental constraints using the IDPConformer Generator (Teixeira et al., 2022). Examples based on AF2 predictions or experimental structures of folded domains can be found in PED entries 435, 436, 437, and 438.

We have defined a new single‐valued measure for the similarity of two ensemble structures of the same protein. This measure can be used to detect differences between states of a protein as well as between models generated using different approaches. Two site‐specific disorder measures reveal subtle differences in the conformation of chain segments and weak local order. The deviation of IDPs from Flory random coil behavior can be used to detect weak order. We have introduced a deviation matrix that reveals heterogeneous compaction and extension along the peptide chain of IDPs or IDRs.

The field of ensemble modeling and analysis is still far from mature. Open questions include the sufficient sampling of large conformational spaces, the proper relative weighting of partially inconsistent restraint sets, the optimal size of a representative ensemble, and the separation of ensemble width in conformational space from model uncertainty (Bottaro & Lindorff‐Larsen, 2018; Jeschke, 2022). Progress in these directions depends on improved experimental design and tools for ensemble analysis and comparison. We hope that MMMx can contribute to these efforts. MMMx can be downloaded from github.com/gjeschke/MMMx and its documentation is available at mmmx.info.

## 4 METHODS

### 4.1 Domain partitioning

We downloaded the predictions for the human proteome from the AF‐PSD (Jumper et al., 2021; Tunyasuvunakool et al., 2021) at alphafold.ebi.ac.uk/download and compiled a list of file names. For each file in the list, we downloaded the PAE matrix ***M*** and sequence information via the database API. We discarded entries of proteins with <50 residues and wrote out the sequences in FASTA format for processing with eSpritz (Walsh et al., 2012). For further processing, we symmetrized ***M*** and converted it into a binary matrix ***B***. To do this, we set the entries to one if the PAE was below the domain threshold *t*_domain_ and to zero otherwise. For each residue with index *i*, we determined the maximal value *e*_*i*_ for which all matrix elements *B*_*ij*_ with *j* = *i* + 1… *i* + Δ*N* were one. We arranged the *e*_*i*_ in a vector ***e*** and applied a moving average with window width 5 (local context) to ***e*** and rounded to integer elements. Local maxima in ***e*** are expected to correspond to the approximate size of domains. With the maximum *e*_max_ of ***e***, we defined a preliminary folded domain (*i*_max(***e***)_, *i*_max(***e***)_ + *e*_max_), where *i*_max(***e***)_ is the residue where the maximum occurs. We refined domain boundaries by adding residues for which the mean PAE to all residues already in the domain was below *t*_domain_. We then set all elements of ***e*** belonging to the domain to zero and all elements of ***M*** to a very large number (10^6^). This procedure is repeated as long as *e*_max_ is larger than or equal to the minimum domain size (default 25). We combined folded domains that were separated by a linker of less than three residues. As a default value of *t*_domain_, we use a third of the value where PAE is capped (31.75 Å). By visually inspecting the domain partitioning versus the PAE matrix for the first 500 proteins in the AF2 prediction of the human proteome, we confirmed that this default value *t*_domain_ = 10.58 Å resulted in a plausible domain partitioning. We obtained the uncertainty estimates in Table 1 (95% confidence intervals) by varying *t*_domain_ between 8.58 and 12.58 Å in steps of 0.1 Å.

For benchmarking, we processed the entire set of FASTA sequences for the AF2 predictions of the human proteome on the eSpritz server with the default prediction type “x‐ray” and the decision threshold “Best Sw.” We assigned folded domains as consecutive stretches of at least 25 ordered residues, defined as residues with a disorder parameter below a threshold *t*_disorder_. Again, we combined domains that were separated by a linker of less than three residues. The uncertainty estimates in Table 1 (95% confidence intervals) was obtained by varying *t*_disorder_ between 0.14 and 0.16 in steps of 0.001. For the eight selected proteins in Figures 3 and S2, the predictions of SETH (Ilzhöfer et al., 2022) were determined using its Colab implementation. We defined residues with CheZOD ≥8 as ordered, and assigned the domains in the same way as for the eSpritz predictions.

### 4.2 Similarity analysis

We downloaded the metadata for all PED entries via the API and identified entries for which more than one ensemble was stored. From these entries, we discarded those with ensemble sizes >1000 conformers. For each of the remaining entries, we computed the pairwise similarity measures *s*_*kl*_ between the ensembles of the same entry according to Equation (4). For the case with the lowest *s*_*kl*_ = 0.449 (ensembles 2 and 4 of PED00020), we performed a detailed comparison (see Supporting information S1).

We compiled a list of PED entries for the N‐terminal targeting domain of yeast Sic1 (Table S1) and calculated pairwise similarity measures *s*_*kl*_ according to Equation (4) between all ensembles deposited under these entries.

### 4.3 Visualization of IDPs

For each conformer in an ensemble, we calculate the inertia tensor

(15) $$I=\sum_{a=1}^{A}m_{a}\left(\begin{array}{ccc} y_{a}^{2}+z_{a}^{2} & -x_{a}y_{a} & -x_{a}z_{a}\\ -x_{a}y_{a} & x_{a}^{2}+z_{a}^{2} & -y_{a}z_{z}\\ -x_{a}z_{a} & -y_{a}z_{a} & x_{a}^{2}+y_{a}^{2} \end{array}\right),$$

where the index *a* runs over the *A* atoms of the conformer, the *m*_*a*_ are the atomic masses and the (*x*_*a*_, *y*_*a*_, *z*_*a*_) are the Cartesian atomic coordinates. We diagonalize this tensor and thus obtain the eigenvalues *I*_*x*_, *I*_*y*_, *I*_*z*_, ordered by increasing moment of inertia, as well as the eigenvectors. We arrange the three eigenvectors in a rotation matrix, which we use to transform all atomic coordinates into the PAS of ***I***. If the first atom of the N‐terminal residue has a larger *x*‐ or *z*‐coordinate than the last atom of the C‐terminal residue, we reverse the signs of two of the Cartesian coordinates to position the N‐terminus at low *x* and *z*.

To create a space‐filling model of the ensemble, we calculate a pseudo‐electron density by weighted addition of the simulated electron densities of the individual conformers. We calculate the electron density maps of each conformer using an algorithm previously introduced for MD simulations (Briones et al., 2019). By default, we display an isosurface that encompasses 99.9% of the total pseudo‐electron density of the ensemble. To map an approximate electrostatic potential onto the surface, we calculate the charges of the negatively charged side chains from their pKa values and pH (default pH 7.0). For carboxylate side groups we localize the charge at the middle coordinate of the two oxygen atoms and for cationic side groups at the middle coordinate of the relevant nitrogen atoms. For phosphorylated side groups, we assign a charge of −2 and place it at the middle coordinate of the three terminal oxygen atoms of the phosphate group. To calculate the electrostatic potential at the surface of the density map, we assume an exponential decay with a Debye length determined by the ionic strength (default value 150 mM). We assign the surface a color between red (maximum negative) and blue (maximum positive), corresponding to the sum of the electrostatic potentials of all charged side groups. White color corresponds to zero potential. Although this electrostatic model is only crude, we consider it sufficient because an electrostatic potential for an ensemble is only semiquantitative from the outset.

### 4.4 Source code availability, documentation, and computational requirements

The source code of MMMx and the commented scripts to create the figures in this article are included in the MMMx package on GitHub (github.com/gjeschke/MMMx, commit 25e4025, downloaded on November 30, 2023). These scripts directly download AF2 predictions from AF‐PSD and ensembles from PED or Zenodo. For some figures, layout was edited in CorelDraw. We created figures with MMMx, whereas for Figures 4, S4, and S11 we use in addition MMM (github.com/gjeschke/MMM, commit 65f28a7, downloaded November 10, 2023) for visualization. The documentation of MMMx can be found at mmmx.info. The English style in this manuscript has been improved by a DeepL English–German–English cycle with manual corrections to the German and final English text where necessary.

Most of the examples in this article were computed on a single core of a desktop PC running Matlab 2021 or 2022 (The MathWorks, Inc.) under Windows 10 or Ubuntu Linux. The Rigi and Flex modules for ensemble modeling are parallelized. For the generation of large raw ensembles with these modules, the use of a computer cluster is recommended. The unconstrained ensemble of FUS NTD, which was used as a reference for the spatially resolved order and flexibility parameters (Figure S6), was computed on the Euler cluster of ETH Zürich. As an example for computing time requirements, we generated an unrestrained ensemble of full‐length SRSF1 (248 residues, 2 folded domains, 33 residues long linker between domains, 53 residues long C‐terminal IDR) by the RigiFlex approach. We started with 50,000 trials for rigid‐body arrangement and reduced the number of such arrangements to 2000 by hierarchical clustering. Flexible linkers between the folded domains could be generated for 465 arrangements. Generation of the C‐terminal IDR succeeded for all 465 conformers. The whole computation took 136 h 39 min on an Intel(R) Core(TM) i7‐9700K CPU @ 3.60 GHz with 8 cores. Out of this time, 123 h 6 min were used for generation of the flexible linkers between the folded domains and 44 min (5.7 s/conformer) for generation of the C‐terminal IDRs.

## AUTHOR CONTRIBUTIONS

**Gunnar Jeschke:** Conceptualization; methodology; software; validation; investigation; formal analysis; project administration; visualization; writing—original draft; funding acquisition; writing—review and editing; resources.

## Supporting information


**Data S1:** Supporting information.

## References

[pro4906-bib-0001] Aina A , Hsueh SCC , Plotkin SS . PROTHON: a local order parameter‐based method for efficient comparison of protein ensembles. J Chem Inf Model. 2023;63:3453–3461. 10.1021/acs.jcim.3c00145 37178169

[pro4906-bib-0002] Alston JJ , Ginell GM , Soranno A , Holehouse AS . The analytical Flory random coil is a simple‐to‐use reference model for unfolded and disordered proteins. J Phys Chem B. 2023;127:4746–4760. 10.1021/acs.jpcb.3c01619 37200094 PMC10875986

[pro4906-bib-0003] Best RB . Emerging consensus on the collapse of unfolded and intrinsically disordered proteins in water. Curr Opin Struct Biol. 2020;60:27–38. 10.1016/j.sbi.2019.10.009 31805437 PMC7472963

[pro4906-bib-0004] Bonomi M , Camilloni C . Integrative structural and dynamical biology with PLUMED‐ISDB. Bioinformatics (Oxford, England). 2017;33(24):3999–4000. 10.1093/bioinformatics/btx529 28961689

[pro4906-bib-0005] Bonomi M , Camilloni C , Cavalli A , Vendruscolo M . Metainference: a Bayesian inference method for heterogeneous systems. Sci Adv. 2016;2:e150117. 10.1126/sciadv.1501177 PMC4737209 26844300

[pro4906-bib-0006] Bottaro S , Lindorff‐Larsen K . Biophysical experiments and biomolecular simulations: a perfect match? Science. 2018;361:355–360. 10.1126/science.aat4010 30049874

[pro4906-bib-0007] Briones R , Blau C , Kutzner C , de Groot BL , Aponte‐Santamaría C . GROmaρs: a GROMACS‐based toolset to analyze density maps derived from molecular dynamics simulations. Biophys J. 2019;116:4–11. 10.1016/j.bpj.2018.11.3126 30558883 PMC6342704

[pro4906-bib-0008] Brüschweiler R . Efficient RMSD measures for the comparison of two molecular ensembles. Proteins. 2003;50:26–34. 10.1002/prot.10250 12471596

[pro4906-bib-0009] Conev A , Rigo MM , Devaurs D , Fonseca AF , Kalavadwala H , de Freitas MV , et al. EnGens: a computational framework for generation and analysis of representative protein conformational ensembles. Brief Bioinform. 2023;24:bbad242. 10.1093/bib/bbad242 37418278 PMC10359083

[pro4906-bib-0010] Conte AD , Mehdiabadi M , Bouhraoua A , Miguel Monzon A , Tosatto SCE , Piovesan D . Critical assessment of protein intrinsic disorder prediction (CAID) – results of round 2. Proteins. 2023;91:1925–1934. 10.1002/prot.26582 37621223

[pro4906-bib-0011] Dorn G , Gmeiner C , de Vries T , Dedic E , Novakovic M , Damberger FF , et al. Integrative solution structure of PTBP1‐IRES complex reveals strong compaction and ordering with residual conformational flexibility. Nat Commun. 2023;14:6429. 10.1038/s41467-023-42012-z 37833274 PMC10576089

[pro4906-bib-0012] Esteban‐Hofer L , Emmanouilidis L , Yulikov M , Allain FHT , Jeschke G . Ensemble structure of the N‐terminal domain (1‐267) of FUS in a biomolecular condensate (1.1) [data set]. Zenodo. 2023 10.5281/zenodo.8214049 38279531

[pro4906-bib-0013] Eustermann S , Wu W‐F , Langelier M‐F , Yang J‐C , Easton LE , Riccio AA , et al. Structural basis of detection and signaling of DNA single‐strand breaks by human PARP‐1. Mol Cell. 2015;60:742–754. 10.1016/j.molcel.2015.10.032 26626479 PMC4678113

[pro4906-bib-0014] Fu H , Shao X , Cai W , Chipot C . Taming rugged free energy landscapes using an average force. Acc Chem Res. 2019;52:3254–3264. 10.1021/acs.accounts.9b00473 31680510

[pro4906-bib-0015] Fuertes G , Banterle N , Ruff KM , Chowdhury A , Mercadante D , Koehler C , et al. Decoupling of size and shape fluctuations in heteropolymeric sequences reconciles discrepancies in SAXS vs. FRET measurements. Proc Natl Acad Sci U S A. 2017;114:E6342–E6351. 10.1073/pnas.1704692114 28716919 PMC5547626

[pro4906-bib-0016] Gama Lima Costa R , Fushman D . Reweighting methods for elucidation of conformation ensembles of proteins. Curr Opin Struct Biol. 2022;77:102470. 10.1016/j.sbi.2022.102470 36183447 PMC9771963

[pro4906-bib-0017] Ghafouri H , Lazar T , Del Conte A , Tenorio Ku LG , Aspromonte MC , Bernadó P , et al. PED in 2024: improving the community deposition of structural ensembles for intrinsically disordered proteins. Nucleic Acids Res. 2023;52:D536–D544. 10.1093/nar/gkad947 PMC10767937 37904608

[pro4906-bib-0018] Gmeiner C , Dorn G , Allain FH‐T , Jeschke G , Yulikov M . Spin labelling for integrative structure modelling: a case study of the polypyrimidine‐tract binding protein 1 domains in complexes with short RNAs. Phys Chem Chem Phys. 2017;19:28360–28380. 10.1039/c7cp05822e 29034946

[pro4906-bib-0019] Gomes G‐N , Krzeminski M , Namini A , Martin EW , Mittag T , Head‐Gordon T , et al. Conformational ensembles of an intrinsically disordered protein consistent with NMR, SAXS, and single‐molecule FRET. J Am Chem Soc. 2020;142:15697–15710. 10.1021/jacs.0c02088 32840111 PMC9987321

[pro4906-bib-0020] González‐Delgado J , Sagar A , Zanon C , Lindorff‐Larsen K , Bernadó P , Neuvial P , et al. WASCO: a Wasserstein‐based statistical tool to compare conformational ensembles of intrinsically disordered proteins. J Mol Biol. 2023;435:168053. 10.1016/j.jmb.2023.168053 36934808

[pro4906-bib-0021] Hovmöller S , Zhou T , Ohlson T . Conformations of amino acids in proteins. Acta Cryst D. 2002;58:768–776. 10.1107/s0907444902003359 11976487

[pro4906-bib-0022] Huang JR , Warner LR , Sanchez C , Gabel F , Madl T , Mackereth CD , et al. Transient electrostatic interactions dominate the conformational equilibrium sampled by multidomain splicing factor U2AF65: a combined NMR and SAXS study. J Am Chem Soc. 2014;136:7068–7076. 10.1021/ja502030n 24734879

[pro4906-bib-0023] Huihui J , Ghosh K . An analytical theory to describe sequence‐specific inter‐residue distance profiles for polyampholytes and intrinsically disordered proteins. J Chem Phys. 2020;152:161102. 10.1063/5.0004619 32357776 PMC7180065

[pro4906-bib-0024] Huihui J , Ghosh K . Intrachain interaction topology can identify functionally similar intrinsically disordered proteins. Biophys J. 2021;120:1860–1868. 10.1016/j.bpj.2020.11.2282 33865811 PMC8204386

[pro4906-bib-0025] Hummer G , Köfinger J . Bayesian ensemble refinement by replica simulations and reweighting. J Chem Phys. 2015;143:243150. 10.1063/1.4937786 26723635

[pro4906-bib-0026] Ilzhöfer D , Heinzinger M , Rost B . SETH predicts nuances of residue disorder from protein embeddings. Front Bioinform. 2022;2:1019597. 10.3389/fbinf.2022.1019597 36304335 PMC9580958

[pro4906-bib-0027] Jeschke G . DEER distance measurements on proteins. Annu Rev Phys Chem. 2012a;63:419–446. 10.1146/annurev-physchem-032511-143716 22404592

[pro4906-bib-0028] Jeschke G . Characterization of protein conformational changes with sparse spin‐label distance constraints. J Chem Theory Comput. 2012b;8:3854–3863. 10.1021/ct300113z 26593026

[pro4906-bib-0029] Jeschke G . Optimization of algorithms for modeling protein structural transitions from sparse long‐range spin‐label distance constraints. Z Phys Chem. 2012c;226:1395–1414. 10.1524/zpch.2012.0289

[pro4906-bib-0030] Jeschke G . Ensemble models of proteins and protein domains based on distance distribution restraints: ensembles by distance distribution restraints. Proteins. 2016;84:544–560. 10.1002/prot.25000 26994550

[pro4906-bib-0031] Jeschke G . MMM : Integrative ensemble modeling and ensemble analysis. Protein Sci. 2021;30:125–135. 10.1002/pro.3965 33015891 PMC7737775

[pro4906-bib-0032] Jeschke G . Integration of nanometer‐range label‐to‐label distances and their distributions into modelling approaches. Biomolecules. 2022;12:1369. 10.3390/biom12101369 36291578 PMC9599366

[pro4906-bib-0033] Jeschke G , Esteban‐Hofer L . Integrative ensemble modeling of proteins and their complexes with distance distribution restraints. Methods Enzymol. 2022;666:145–169. 35465919 10.1016/bs.mie.2022.02.010

[pro4906-bib-0034] Jones S , Stewart M , Michie A , Swindells MB , Orengo C , Thornton JM . Domain assignment for protein structures using a consensus approach: characterization and analysis. Protein Sci. 1998;7:233–242. 10.1002/pro.5560070202 9521098 PMC2143930

[pro4906-bib-0035] Jumper J , Evans R , Pritzel A , Green T , Figurnov M , Ronneberger O , et al. Highly accurate protein structure prediction with AlphaFold. Nature. 2021;596:583–589. 10.1038/s41586-021-03819-2 34265844 PMC8371605

[pro4906-bib-0036] Jussupow A , Kaila VRI . Effective molecular dynamics from neural network‐based structure prediction models. J Chem Theory Comp. 2023;19:1965–1975. 10.1021/acs.jctc.2c01027 PMC11181330 36961997

[pro4906-bib-0037] Lazar T , Guharoy M , Vranken W , Rauscher S , Wodak SJ , Tompa P . Distance‐based metrics for comparing conformational ensembles of intrinsically disordered proteins. Biophys J. 2020;118:2952–2965. 10.1016/j.bpj.2020.05.015 32502383 PMC7300341

[pro4906-bib-0038] Lazar T , Martínez‐Pérez E , Quaglia F , Hatos A , Chemes LB , Iserte JA , et al. PED in 2021: a major update of the protein ensemble database for intrinsically disordered proteins. Nucleic Acids Res. 2021;49:D404–D411. 10.1093/nar/gkaa1021 33305318 PMC7778965

[pro4906-bib-0039] Lindorff‐Larsen K , Ferkinghoff‐Borg J . Similarity measures for protein ensembles. PLoS One. 2009;4:e4203. 10.1371/journal.pone.0004203 19145244 PMC2615214

[pro4906-bib-0040] Lindorff‐Larsen K , Kristjansdottir S , Teilum K , Fieber W , Dobson CM , Poulsen FM , et al. Determination of an ensemble of structures representing the denatured state of the bovine acyl‐coenzyme a binding protein. J Am Chem Soc. 2004;126:3291–3299. 10.1021/ja039250g 15012160

[pro4906-bib-0041] MacArthur MW , Thornton JM . Conformational analysis of protein structures derived from NMR data. Proteins. 1993;17:232–251. 10.1002/prot.340170303 8272423

[pro4906-bib-0042] Mittag T , Marsh J , Grishaev A , Orlicky S , Lin H , Sicheri F , et al. Structure/function implications in a dynamic complex of the intrinsically disordered Sic1 with the Cdc4 subunit of an SCF ubiquitin ligase. Structure. 2010;18:494–506. 10.1016/j.str.2010.01.020 20399186 PMC2924144

[pro4906-bib-0043] Oates ME , Romero P , Ishida T , Ghalwash M , Mizianty MJ , Xue B , et al. D2P2: database of disordered protein predictions. Nucleic Acids Res. 2012;41:D508–D516. 10.1093/nar/gks1226 23203878 PMC3531159

[pro4906-bib-0044] Orioli S , Larsen AH , Bottaro S , Lindorff‐Larsen K . How to learn from inconsistencies: integrating molecular simulations with experimental data. Prog Mol Biol Transl Sci. 2020;170:123–176. 10.1016/bs.pmbts.2019.12.006 32145944

[pro4906-bib-0045] Ozenne V , Bauer F , Salmon L , Huang JR , Jensen MR , Segard S , et al. Flexible‐meccano: a tool for the generation of explicit ensemble descriptions of intrinsically disordered proteins and their associated experimental observables. Bioinformatics. 2012;28:1463–1470. 10.1093/bioinformatics/bts172 22613562

[pro4906-bib-0046] Ozenne V , Schneider R , Yao M , Huang JR , Salmon L , Zweckstetter M , et al. Mapping the potential energy landscape of intrinsically disordered proteins at amino acid resolution. J Am Chem Soc. 2012;134:15138–15148. 10.1021/ja306905s 22901047

[pro4906-bib-0047] Pereira B , Billaud M , Almeida R . RNA‐binding proteins in cancer: old players and new actors. Trends Cancer. 2017;3:506–528. 10.1016/j.trecan.2017.05.003 28718405

[pro4906-bib-0048] Piai A , Calçada EO , Tarenzi T , del Grande A , Varadi M , Tompa P , et al. Just a flexible linker? The structural and dynamic properties of CBP‐ID4 revealed by NMR spectroscopy. Biophys J. 2016;110:372–381. 10.1016/j.bpj.2015.11.3516 26789760 PMC4724632

[pro4906-bib-0049] Polyhach Y , Bordignon E , Jeschke G . Rotamer libraries of spin labelled cysteines for protein studies. Phys Chem Chem Phys. 2011;13:2356–2366. 10.1039/c0cp01865a 21116569

[pro4906-bib-0050] Polyhach Y , Jeschke G . Prediction of favourable sites for spin labelling of proteins. Spectrosc Int J. 2010;24:651–659. 10.1155/2010/706498

[pro4906-bib-0051] Ravera E , Sgheri L , Parigi G , Luchinat C . A critical assessment of methods to recover information from averaged data. Phys Chem Chem Phys. 2016;18:5686–5701. 10.1039/c5cp04077a 26565805

[pro4906-bib-0052] Ritsch I , Esteban‐Hofer L , Lehmann E , Emmanouilidis L , Yulikov M , Allain FHT , et al. Characterization of weak protein domain structure by spin‐label distance distributions. Front Mol Biosci. 2021;8:636599. 10.3389/fmolb.2021.636599 33912586 PMC8072059

[pro4906-bib-0053] Ritsch I , Lehmann E , Emmanouilidis L , Yulikov M , Allain F , Jeschke G . Phase separation of heterogeneous nuclear ribonucleoprotein A1 upon specific RNA‐binding observed by magnetic resonance. Angew Chem Int ed. 2022;61:e202204311. 10.1002/anie.202204311 PMC9804974 35866309

[pro4906-bib-0054] Ruff KM , Pappu RV . AlphaFold and implications for intrinsically disordered proteins. J Mol Biol. 2021;433:167208. 10.1016/j.jmb.2021.167208 34418423

[pro4906-bib-0055] Salmon L , Nodet G , Ozenne V , Yin G , Jensen MR , Zweckstetter M , et al. NMR characterization of long‐range order in intrinsically disordered proteins. J Am Chem Soc. 2010;132:8407–8418. 10.1021/ja101645g 20499903

[pro4906-bib-0056] Schiemann O , Heubach CA , Abdullin D , Ackermann K , Azarkh M , Bagryanskaya EG , et al. Benchmark test and guidelines for DEER/PELDOR experiments on nitroxide‐labeled biomolecules. J Am Chem Soc. 2021;143:17875–17890. 10.1021/jacs.1c07371 34664948 PMC11253894

[pro4906-bib-0057] Teixeira JMC , Liu ZH , Namini A , Li J , Vernon RM , Krzeminski M , et al. IDPConformerGenerator: a flexible software suite for sampling the conformational space of disordered protein states. J Phys Chem A. 2022;126:5985–6003. 10.1021/acs.jpca.2c03726 36030416 PMC9465686

[pro4906-bib-0058] Tiberti M , Papaleo E , Bengtsen T , Boomsma W , Lindorff‐Larsen K . ENCORE: software for quantitative ensemble comparison. PLoS Comp Biol. 2015;11:e1004415. 10.1371/journal.pcbi.1004415 PMC4624683 26505632

[pro4906-bib-0059] Tunyasuvunakool K , Adler J , Wu Z , Green T , Zielinski M , Žídek A , et al. Highly accurate protein structure prediction for the human proteome. Nature. 2021;596:590–596. 10.1038/s41586-021-03828-1 34293799 PMC8387240

[pro4906-bib-0060] Vitali F , Henning A , Oberstrass FC , Hargous Y , Auweter SD , Erat M , et al. Structure of the two most C‐terminal RNA recognition motifs of PTB using segmental isotope labeling. EMBO J. 2006;25:150–162. 10.1038/sj.emboj.7600911 16362043 PMC1356354

[pro4906-bib-0061] Vögele M , Thomson NJ , Truong ST , McAvity J , Zachariae U , Dror RO . Systematic analysis of biomolecular conformational ensembles with PENSA. 2022 10.48550/ARXIV.2212.02714 PMC11698571 39745157

[pro4906-bib-0062] Walsh I , Martin AJM , Di Domenico T , Tosatto SCE . ESpritz: accurate and fast prediction of protein disorder. Bioinformatics. 2012;28:503–509. 10.1093/bioinformatics/btr682 22190692

[pro4906-bib-0063] Zhang S , Krieger JM , Zhang Y , Kaya C , Kaynak B , Mikulska‐Ruminska K , et al. ProDy 2.0: increased scale and scope after 10 years of protein dynamics modelling with python. Bioinformatics. 2021;37:3657–3659. 10.1093/bioinformatics/btab187 33822884 PMC8545336

[pro4906-bib-0064] Zheng W , Brooks BR . Modeling protein conformational changes by iterative fitting of distance constraints using reoriented normal modes. Biophys J. 2006;90:4327–4336. 10.1529/biophysj.105.076836 16565046 PMC1471861

