# Supplementary material for: Protein ensemble modeling and analysis with MMMx
Source: Protein Sci. 2024 Feb 15;33(3):e4906. doi: 10.1002/pro.4906 (PMC10868441; doi:10.1002/pro.4906)
Supplement: Supplementary file 1 — Data S1: Supporting information. [file PRO-33-e4906-s001.pdf]

Supporting Information

Protein Ensemble Modelling and Analysis with MMMx

G. Jeschke*[1]

[1]ETH Zürich, Department of Chemistry and Applied Biosciences, Vladimir-Prelog-Weg 2, 8093 Zürich, Switzerland

January 4, 2024

S1 Installing and running MMMx

S1.1 Installation

The source-code version of MMMx can be downloaded from https://github.com/gjeschke/MMMx. It is tested on Matlab R2019b (The MathWorks Inc., Natick, Massachusetts) and later Matlab versions. All scripts for generating figures and tables were tested with commit 25e4025, downloaded on November 30, 2023. We recommend using a Git source code management program for convenient update of MMMx to the newest version. Otherwise, the package can also be downloaded as a zipped archive from GitHub. The directory of MMMx with all subdirectories needs to be included in the Matlab path. For structure visualization (Figure S5), MMM needs to be downloaded from https://github.com/gjeschke/MMM and included with all its subdirectories in the Matlab path as well. Tests were run with MMM commit 65f28a7 downloaded November 10, 2023. The on-line documentation of MMMx can be found at https://mmmx.info.

*corresponding author, gjeschke@ethz.ch

S1.2 Running MMMx scripts

The MMMx scripts for generating the Figures and Supplementary Figures of the paper as well as the Matlab script that generate the data for Table 1 are distributed in subdirectory \example_set\Protein_Science_Tools_2024. The MMMx scripts can be run at the Matlab prompt by the command `MMMx` *`scriptname`*, where *`scriptname`* stands to the name of the script. The command `MMMx` without argument opens a file browser for selecting a script file.

The scripts `FigureS4a.mcx`, `FigureS4b.mcx`, and `FigureS4c.mcx` require that MMM runs in the same Matlab instance. For this, use the command `MMM` before running these scripts. After rendering a complex visualization including transparency, as is the case here, it can take time until MMM closes.

The figures in the paper and in this Supplementary Material were prepared from the PDF output of MMMx. For Figure S6(a), the vertical axis was adjusted to the range 0.185 to 0.195. For some figures, font sizes or labels were adjusted in order to conform with formatting recommendations of the journal. In most cases, figure titles were adjusted and in some cases, labels were added to axes.

Figures 3, 6, and 7 in the main text as well as S3 and S7 in the Suporting Informations were generated by Matlab scripts (.m). For Figures 3 and S3, the Matlab scripts generate one panel per run. The panel can be selected by editing the first line of the script. The data for Tables 1 and 2 were generated by Matlab scripts as well. In this case, script `Table1_AF2_PAE.m` needs to be run first. This run generates, among else, a file `AF2_proteome_sequences.fasta`. This file needs to be uploaded to the eSpritz server at `http://old.protein.bio.unipd.it/espritz/` and run. After downloading the results and unzipping the file in the same folder, script `Table1_eSpritz.m` provide the data for the second line of Table 1. The script `Table2.m` requires that MMMx script `Figure_S6.mcx` has been processed before, because it generates the input data on site-specific flexibility and order for the unrestrained ensemble of FUS NTD. The raw data for Figure S5 are generated by MMMx script `Figure_S5_data.mcx` and can be found in the log file. The compiled data are included in file `hnRNPA1_similarities.dat`, so Matlab script `Figure_S7.m` can be run independently. Before running the Matlab script `figure_S8.m`, which makes panels (a) and (b) of Figure S8, MMMx script `Figure_S8_S9.mcx` needs to be run to generate the input data files of the Matlab script. Figure panel S5(a) was generated by importing the ensemble with MMMx function `get_ensemble.m` and processing the entity with MMMx function `aligned_uncertainty.m`.

S2 Supplementary Table

Table S1: Comparison of Sic1 ensembles. States are unphosphorylated (U), phsophorylated at residues 5, 33, 45, 69, 76, 80 (P1), phosphorylated at residues 2, 5, 33, 45, 69, 76, 80 (P2), and P1 in complex with SKP1 and CDC4 (C). Methods are NMR chemical shifts (CS), NMR paramagnetic relaxation enhancement (PRE), NMR residual dipolar couplings (RDC), small-angle x-ray scattering (SAXS), and single-molecule Förster resonance energzy transfer (smFRET).

PED ID	ensemble	State	Method	R_g (Å)
23	1	U	RDC, CS, PRE, SAXS	30.99
	2			30.00
	3			29.84
159	1	U	CS, PRE, SAXS	27.55
160	1	U	CS, PRE, SAXS, smFRET	27.81
424	1	U	IDPConformerGenerator, CS	27.48
423	1	U	IDPConformerGenerator	27.23
455	1	U	idpGAN machine learning	25.86
487	1	U	idpGAN machine learning	24.96
1	1	P1	RDC, CS, PRE, SAXS	26.74
	2			26.71
	3			28.15
486	1	P1	idpGAN machine learning	24.35
161	1	P2	CS, PRE, SAXS, smFRET	29.36
454	1	P2	idpGAN machine learning	24.11
14	1	C	RDC, CS, PRE, SAXS	27.97
	2			27.93
	3			27.65

S3 Supplementary Figures

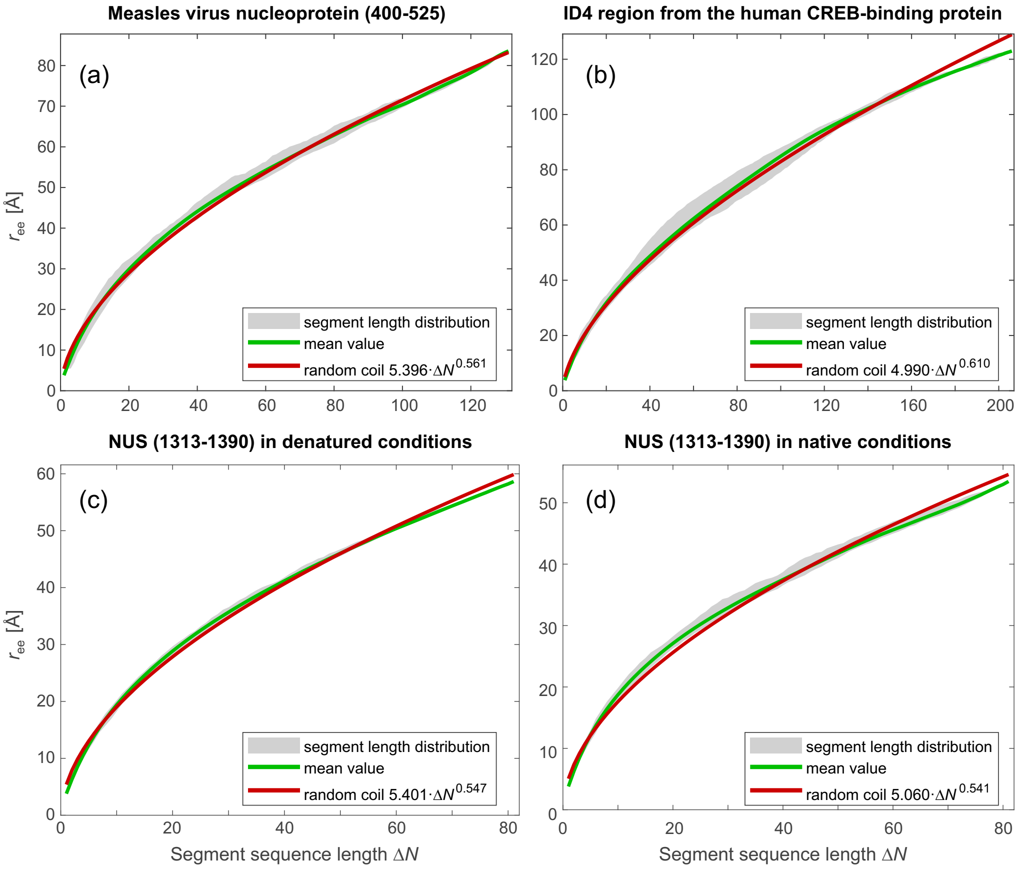

Figure S1: Plot of the distributions of segment root mean square end-to-end distances (grey area) vs. segment sequence length ΔN and their deviation from a random-coil model for (a) measles virus nucleoprotein (PED00020, e001), (b) ID4 region from the human CREB-binding protein (PED00216) (c) NUS (1313-1390) in denatured conditions (PED00149, e001) (d) NUS (1313-1390) in native conditions (PED00150, e001). The green line denotes the mean values for given ΔN, whereas the red line is a fit to a scaling law expected for random coils. The fit parameters are given in the inset.

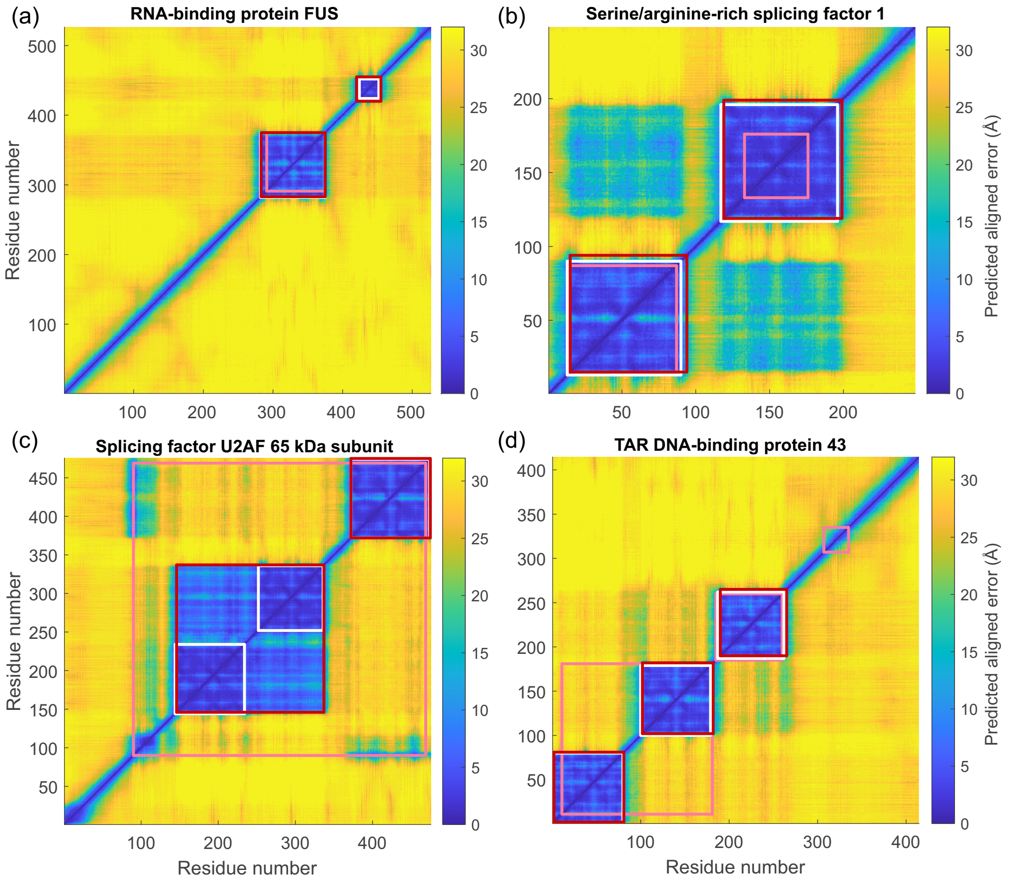

Figure S2: Domain partitioning and classification of proteins by MMMx based on predicted aligned error (PAE) output by AlphaFold2. Recognized domains are marked by red squares. Domain partitioning on the basis of disorder predictions by SETH (white squares) and eSpritz (pink squares) is shown for comparison. (a) RNA-binding protein fused in sarcoma (UniProt P35637) is correctly recognized to feature two folded domains. (b) Serine/arginine-rich splicing factor SRSF1 (UniProt Q07955) is correctly recognized to feature two weakly interacting folded domains. (c) Splicing factor U2AF (UniProt P263689) is correctly recognized to feature three folded domains. (d) TAR DNA binding protein (UniProt Q13148) is recognized to feature three folded domains.

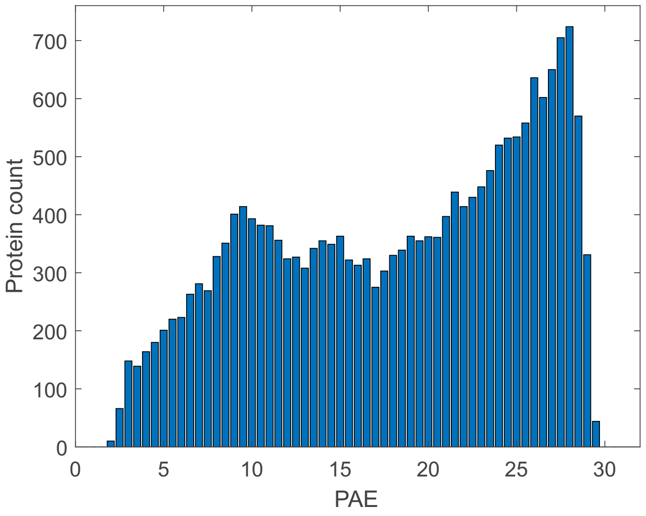

Figure S3: Distribution of mean PAE in the human proteome. PAE values between residues with a sequence distance below 10 were not included in the computation of mean PAE.

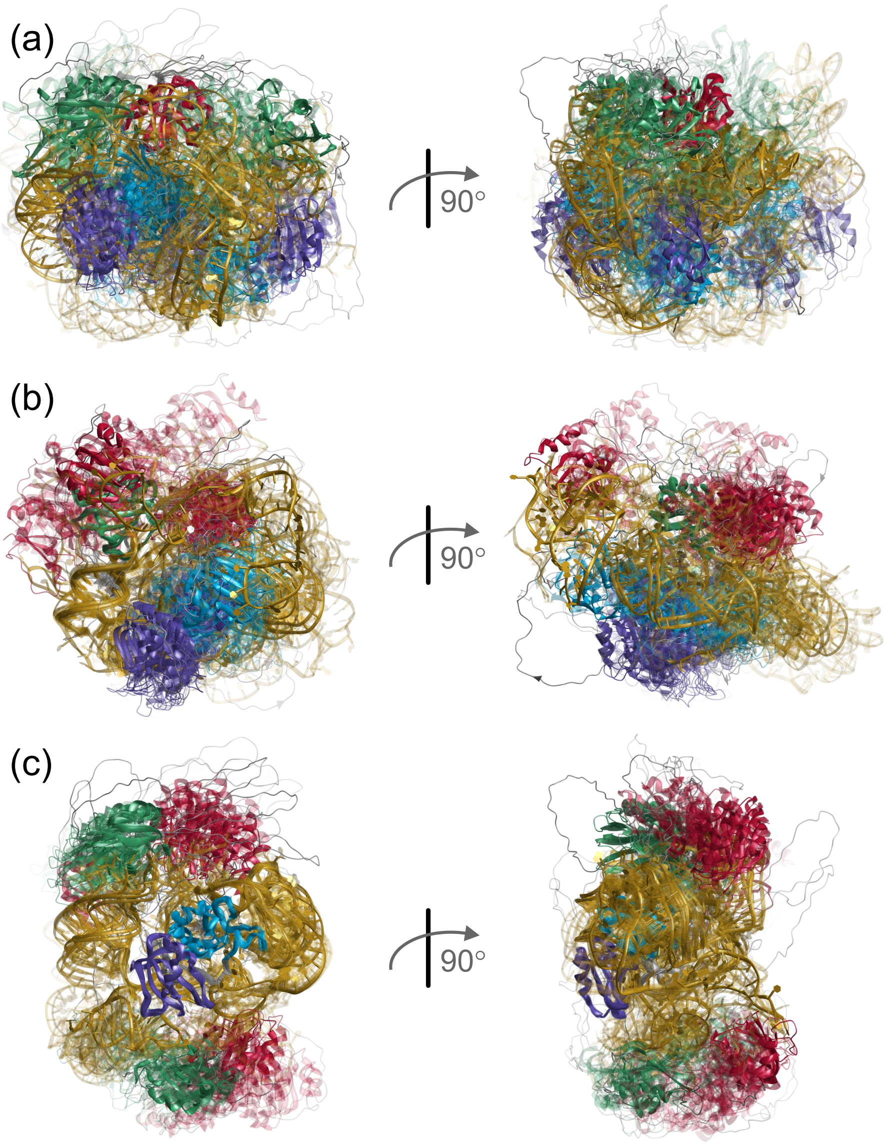

Figure S4: Visualization of an ensemble structure of pyrimidine tract binding protein 1 (PTBP1) in complex with stemlops D to F of the internal ribosome entry site (IRES) of encephalomyocarditis virus (EMCV). RRM1 is shown in crimson color, RRM2 in seagreen color, RRM2 in sky blue color, and RRM 4 in slate blue color. The RNA is shown in gold color and flexible linkers in grey color. Conformer weight is visualized by transparency, with maximum weight corresponding to a fully opaque ribbon model. **(a)** Conformers superimposed on RRM1. **(b)** Conformers superimposed on RRM2. **(c)** Conformers superimposed on RRM3/4.

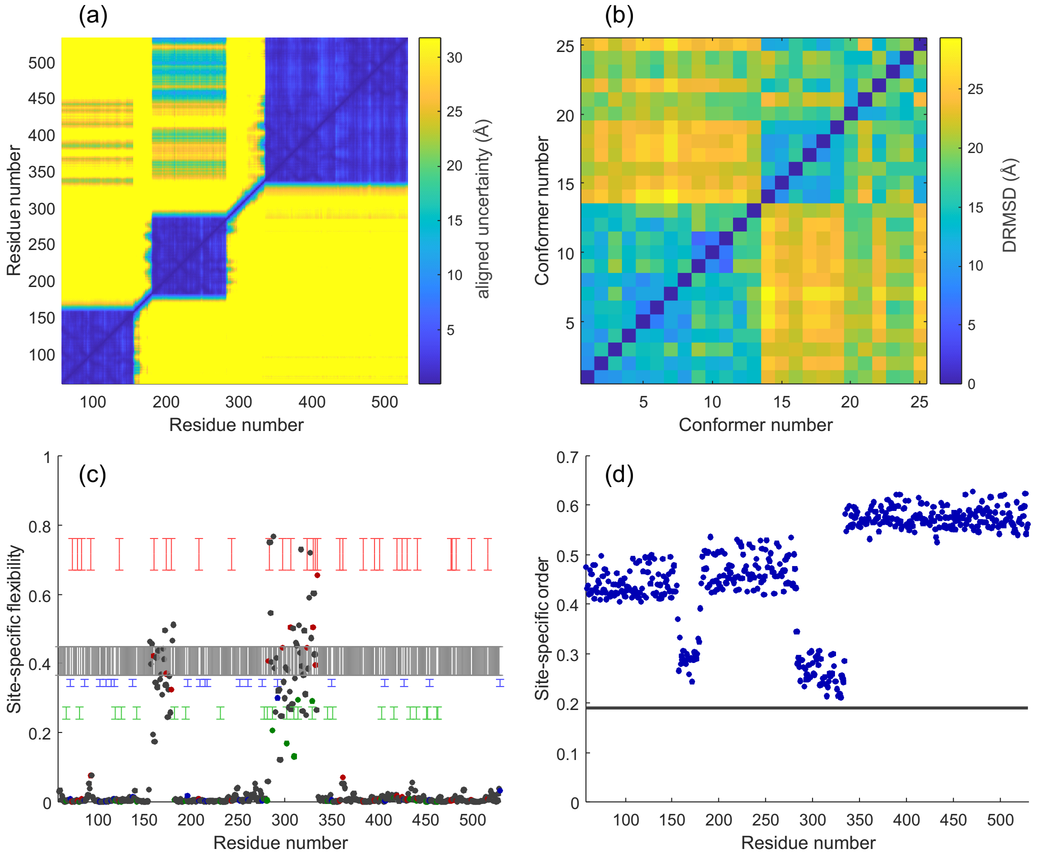

Figure S5: Analysis of an ensemble structure of pyrimidine tract binding protein 1 (PTBP1) in complex with stemlops D to F of the internal ribosome entry site (IRES) of encephalomyocarditis virus (EMCV). **(a)** Aligned uncertainty matrix that is analogous to the AlphaFold2 PAE matrix. (b) DRMSD matrix for the 25 conformers in the ensemble, revealing the existence of two subensembles. (c) Site-specific flexibility clearly reveals the IDRs that link the folded domains. (d) Site=specific order drops to nearly random-coil values in the IDR linkers.

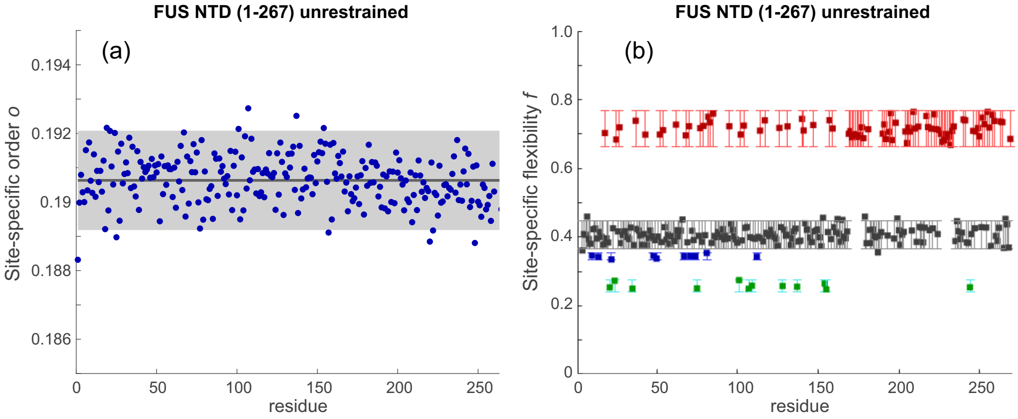

Figure S6: Analysis of the unrestrained ensemble of the N-terminal domain (NTD, 1-267) of fused in sarcoma (FUS). The construct contains three additional residues (1-3), so that the actual FUS NTD corresponds to residue numbers 4-270. (a) Site-specific order parameter versus residue number. Blue dots correspond to per-residue data, the grey horizontal line to the mean value for the whole chain, and the light grean area to the 95% confidence interval. (b) Site-specific flexibility versus residue number. Red symbols correspond to Gly, green Symbols to Pro, blue symbols to Thr, and black symbols to all other residue types. Error bars are centered at the mean value for all residues of this type and correspond to 95% confidence intervals.

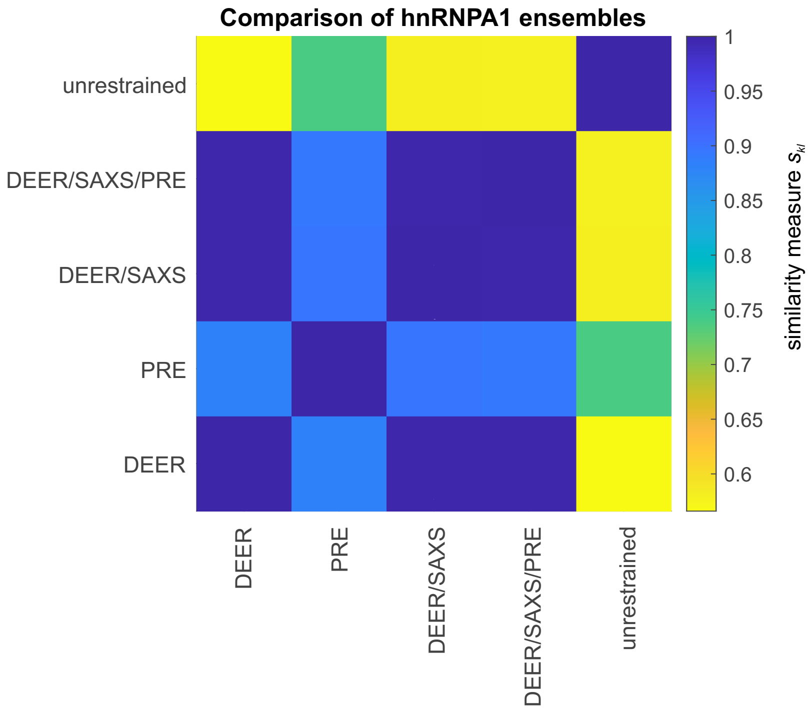

Figure S7: Similarity of ensembles of the C-terminal Gly-rich IDR of hnRNP A1 (187-320). The ensembles were obtained with only DEEER disstance distribution restraints (DEER) only NMR paramagnetic relaxation enhancemebt restraints (PRE), DEER and small-angle x-ray scattering restraints (DEER/SAXS), DEER, PRE, and SAXS testraints (DEER/SAXS/PRE) and without experimental restraints (unrestrained).

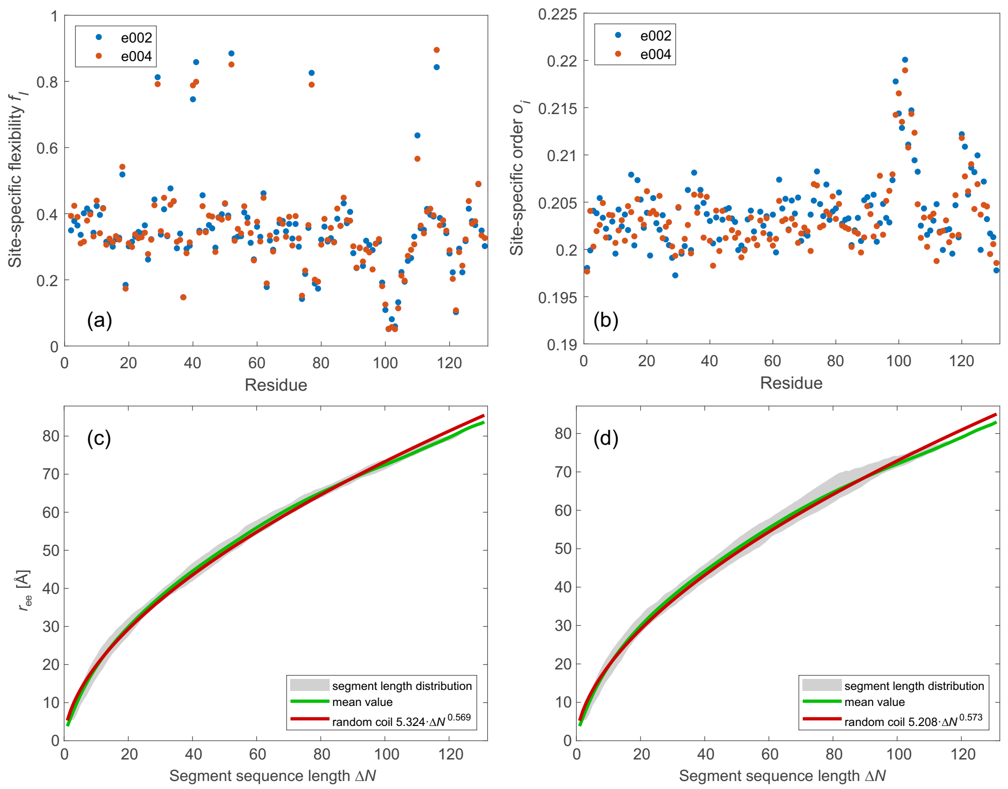

Figure S8: Comparison of ensembles 2 and 4 of PED entry 20 for measles virus nucleoprotein. (a) Site-specific flexibility parameter f_i. (b) Site-specific order parameter o_i. (c) Distribution of section root mean square end-to-end distances e_{ee} (grey areas) and fit by an analytical random-coil model for ensemble 2. Fit parameters are given in the inset. (d) Same as (c), but for ensemble 4.

S4 Ensemble comparison for measles virus nucleoprotein

According to the similarity measure defined in Eq. (4) of the main text, ensembles 2 and 4 of structure PED00020 of measles virus nucleoprotein are rather dissimilar with $s_{kl} = 0.449$. According to analyses of the ensembles in the PED, they have very virtually the same Ramachandran plots, as expected from their agreement with the same NMR data, similar radii of gyration (32.65 and 32.43 Å, respectively), and similar solvent accessibility. Here we investigate the origin of the difference in s_{kl}, which is atypical for ensembles fitted to the same experimental data.

First we consider the site-specific flexibility and order parameters (Figure

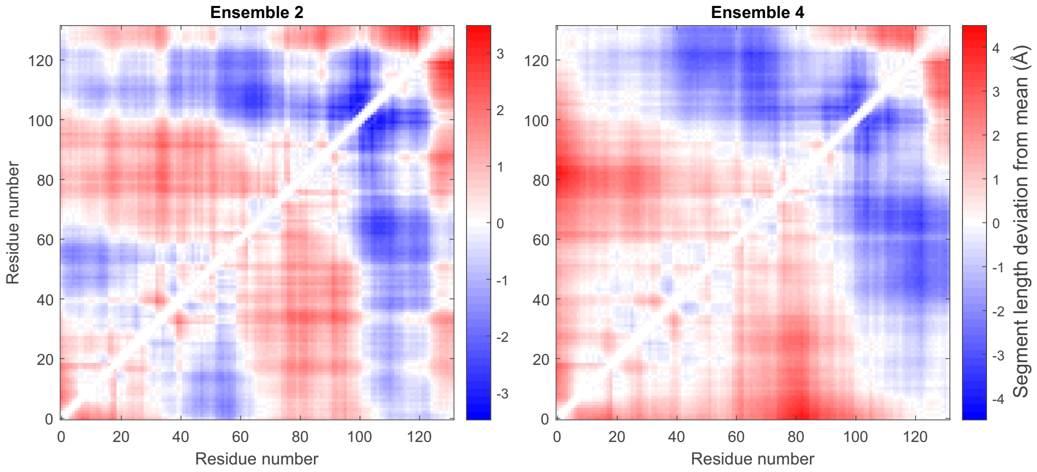

Figure S9: Comparison of ensembles 2 and 4 of PED entry 20 for measles virus nucleoprotein by section length deviation from average root mean square end-to-end distance of all sections of the same length. (a) Ensemble 2. (b) Ensemble 4.

S8(a,b)). The flexibility parameter should be well restrained by the NMR data and is related to the Ramachandran plots. Indeed, we find good agreement of f_i between the two ensembles. The site-specific order parameter may be sensitive to a longer range than NMR data, but also agree quite well between the two ensembles. As might be expected from the very similar radii of gyration, random coil fits (Figure S8(c,d)) do not differ much either. Ensemble 4 (Figure S8(d)) shows a slightly larger variation of section root mean square end-to-end distances, corresponding to slightly larger heterogeneity of chain extension along the sequence. This is borne out by 2D plots of the section-length deviation matrix $\boldsymbol{S}$ (Figure S9). These plots also show a different distribution of extended and compacted sections along the chain, but the effect is not strong.

We then considered pairwise DRMSD between all 400 conformers in the two ensembles, which is closely related to the similarity measure s_{kl}. As an Euclidean distance, DRMSD lends itself to clustering pf conformers. Using the `cluster_transition.m` function of MMMx, we performed hierarchical clustering of the 400 conformers by using $\boldsymbol{D}$ as a distance matrix. We requested nine clusters and found that all clusters are pure in the sense that they contain either only conformers from ensemble 2 or only clusters from ensemble 4 (Figure S10). Clearly, the conformers in ensembles 2 and 4 are different regarding their shape.

To obtain more insight into this difference, we superimposed conformers

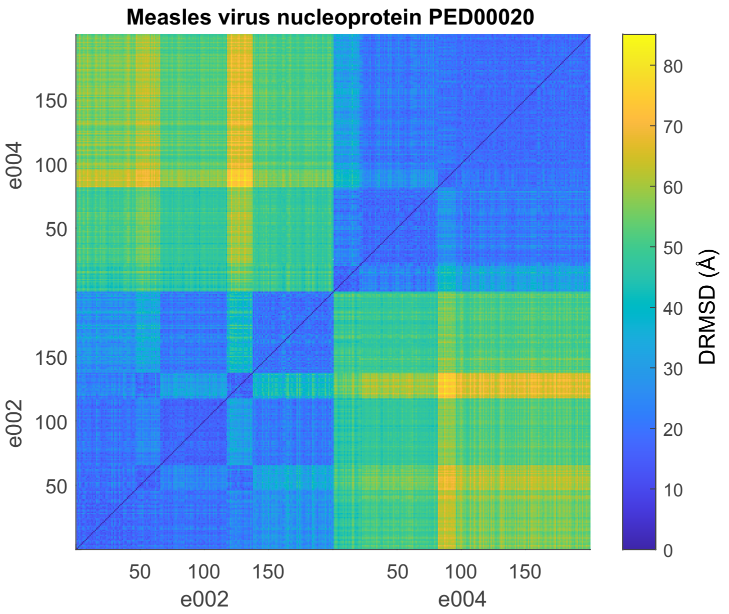

Figure S10: Hierarchical clustering of conformers from ensemble 2 (e002) and ensemble 4 (e004) of measles virus nucleoprotein (PED entry 20). Clusters were ordered such that similarity between neighboring clusters is maximized. Coloring is by pairwise distance root mean square deviation between conformers. The nine clusters can be recognized as blue-shaded squares along the diagonal.

in the eigenframe of their inertia tensors and displayed the two ensembles as coil models with a rainbow color scale starting from blue at the N terminus to red at the C terminus (Figure S11). Indeed, the shape of the two ensembles differs. With the x axis corresponding to the smallest eigenvalue of the inertia tensor upright, ensemble 4 appears more slim than ensemble 2 in both the viewing direction along $-y$ (left panels for each ensemble) and along $-z$ (right panels). On average, the conformers in ensemble 2 are more straight and the ones in ensemble 4 more curved. We tentatively assign this outcome to different possible solutions for fitting the combined shape and alignment of conformers to residual dipolar coupling NMR data.

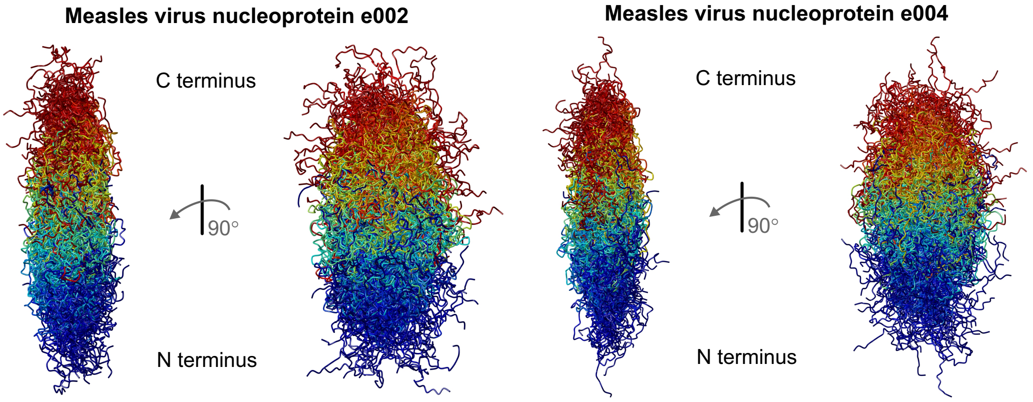

Figure S11: Coil models of ensembles 2 (top) and 4 (bottom) of measles virus nucleoprotein (PED entry 20). Atom coordinates were transformed into the eigenframe of the inertia tensor for each conformer. The such superimposed conformers are displayed with a raimbow color scale along the chain staring with blue at the N terminus and ending with red at the C terminus.
